# Recent advances in non-conventional synthesis of N-heterocyclic compounds: emerging strategies and biological perspectives

**DOI:** 10.1039/d5ra06028a

**Published:** 2025-09-25

**Authors:** Niharan Sivaraj, Kokila Sakthivel, Kotaro Kikushima, Marina D. Kostić, Toshifumi Dohi, Fateh V. Singh

**Affiliations:** a Chemistry Department, SAS, Vellore Institute of Technology – Chennai Vandalur-Kelambakkam Road Chennai 600127 Tamil Nadu India fatehveer.singh@vit.ac.in; b College of Pharmaceutical Sciences, Ritsumeikan University 1-1-1 Nojihigashi Kusatsu Shiga Japan td1203@ph.ritsumei.ac.jp; c Department of Science, Institute for Information Technologies, University of Kragujevac Jovana Cvijića bb 34000 Kragujevac Serbia mrvovic@kg.ac.rs

## Abstract

Nitrogen-containing heterocycles are of particular research interest as they are commonly found in naturally occurring bioactive molecules. However, traditional synthetic approaches to these compounds have various drawbacks, including slow reaction rates, harsh reaction conditions (*e.g.*, high temperatures or strong acids), and low product yields. In recent years, non-conventional synthetic methods, such as microwave irradiation, sonochemical synthesis, and mechanochemical approaches, have emerged as efficient and sustainable alternatives. These techniques provide multiple benefits in synthetic chemistry, enabling faster reactions, enhanced product yields, and superior reaction selectivities. Moreover, they reduce the reliance on toxic solvents and lower the overall energy requirements, ultimately leading to more sustainable processes. Furthermore, the application of green chemistry principles in the synthesis of N-heterocycles has enhanced their environmental compatibility. This review focuses on recent advancements in non-conventional synthetic strategies for constructing N-heterocyclic compounds. Key scaffolds discussed include pyridines, pyrrolidines, pyrroles, imidazoles, pyrazolines, indoles, pyrazoles and 1,2,3-triazoles, along with their fused analogs. These alternative approaches are noted for their synthetic efficiency and environmentally benign nature. Furthermore, the resulting heterocycles exhibit significant potential as biologically active molecules, particularly in the context of their antimicrobial, anticancer, and antioxidant activities.

## Abstract 2

This review highlights the non-conventional synthetic approaches for synthesizing various biologically active nitrogen-containing heterocycles, which represents rapid, efficient, and environmentally friendly alternatives to traditional techniques.

## Graphical abstract

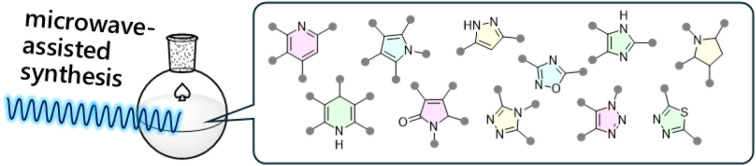

## 1. Introduction

**Fig. 1:**
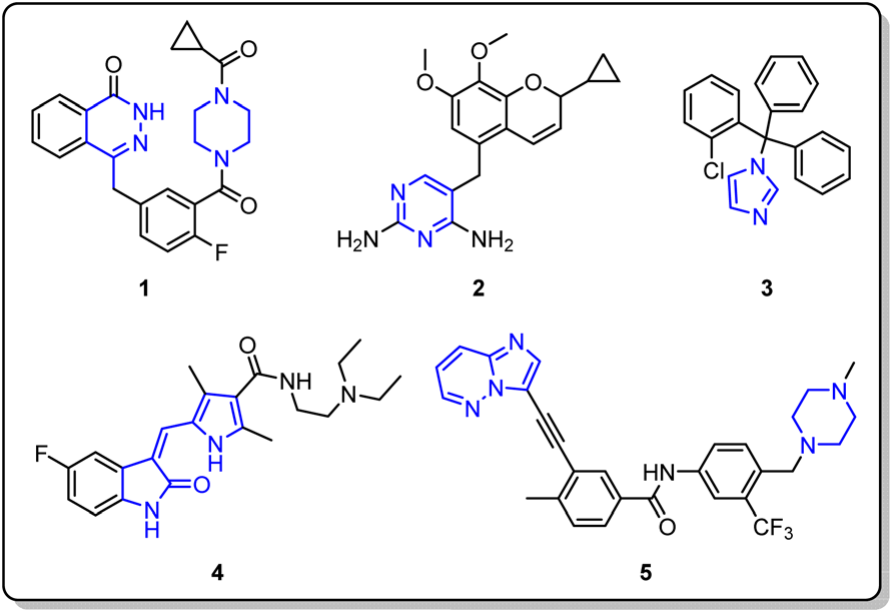
Commercially available drugs containing N-heterocyclic ring systems.

Heterocycles, especially those containing nitrogen atoms, are vital targets in organic synthesis. Indeed, these ring structures have garnered considerable attention due to their prevalence in numerous physiologically active compounds.^1^ A quick analysis of the most potent pharmacophores reveals that N-heterocyclic compounds remain integral in various pharmaceutical fields.^2^ Specifically, they act as scaffolds for molecules with significant biological properties, including antifungal, antibacterial, and anticancer drugs, as well as vitamins and herbicides. Notable examples include olaparib 1, lclaprim 2, clotrimazole 3, sunitinib 4, and ponatinib 5 (Fig. 1).^3–5^

To date, several methods have been developed for synthesizing heterocyclic compounds, which tend to employ expensive resources such as gold, palladium, and other costly materials, along with catalysts that are harmful to the environment.^6–13^ As a result, it is desirable to develop novel and more efficient approaches for the preparation of N-heterocycles using ecologically friendly, atom-economical, and inexpensive catalytic processes.

Although numerous attempts have been made to prepare N-heterocycles, conventional routes remained ineffective.^14–18^ In contrast, non-conventional approaches have been shown to provide a number of benefits over standard strategies. For example, higher yields, enhanced selectivities, and faster reaction rates have been made possible by techniques such as solvothermal synthesis,^19^ microwave-assisted synthesis,^20–25^ sonochemical synthesis,^26–28^ and mechanochemical synthesis.^29^ These techniques also frequently reduce energy consumption, employ fewer solvents, and encourage more sustainable, greener chemical processes. Thus, to overcome the drawbacks associated with traditional methods and improve both productivity and environmental compatibility, non-conventional approaches have gained popularity for the effective and environmentally benign synthesis of bioactive N-heterocycles.

This review therefore aims to highlight the contemporary use of non-conventional protocols for the synthesis of N-heterocycles with potential biological activities *via* one-pot, cycloaddition, and cyclo-condensation approaches, in addition to other modular techniques. The focus of this review is on findings published over the last decade, including the most recent studies up to 2024. Since N-heterocyclic structures exhibit a broad spectrum of biological functions, encompassing anticancer, antiviral, antibacterial, antimicrobial, antineoplastic, and anti-inflammatory effects, among others,^30–34^ many research groups are actively developing new strategies for their preparation, as are outlined below.

### 1.1 Limitations of traditional synthetic approaches for N-heterocycles

Traditional synthetic approaches for N-heterocycles, such as the Skraup, Paal–Knorr, Fischer, Debus–Radziszewski, and Hantzsch reactions, frequently encounter multiple intrinsic drawbacks. Such protocols generally necessitate high temperatures and extended reaction durations, along with the requirement for strong acids, dehydrating substances, or oxidizing agents, ultimately resulting in competitive side reactions, low atom efficiencies, and reduced selectivities.^35,36^ Additionally, the severe reaction conditions required by these approaches frequently limit the substrate scope, particularly when sensitive functional groups are involved, thereby reducing structural variety. In numerous instances, the employment of volatile organic solvents and hazardous chemicals additionally exacerbates the inadequate environmental compatibility and operational safety profiles of these routes.

### 1.2 Microwave-assisted synthesis

Microwave (MW) irradiation is particularly advantageous in the preparation of physiologically relevant N-heterocycles, resulting in higher yields but a lower environmental impact than conventional thermal methods.^37–41^ Because of the homogeneous heat produced by dielectric volumetric heating during MW-assisted organic synthesis (MAOS), reactions tend to occur more quickly and selectively.^42–44^ MW irradiation therefore provides an efficient alternative to conventional heating by directly converting electromagnetic energy into heat, revolutionizing chemistry with faster reactions and improved efficiencies. In the field of organic synthesis, MW irradiation enables shorter reaction times and broader reaction ranges, benefiting various industries (*e.g.*, the pharmaceutical industry) by accelerating the production of novel compounds. MW heating primarily arises from dielectric polarization, where molecules align with oscillating electromagnetic fields, absorbing energy and generating heat. The efficiency of this process is measured by the dissipation factor (tan *δ* = *ε*′′/*ε*′), while the Maxwell–Wagner effect further contributes to an enhanced efficiency in heterogeneous systems.^45^ Polar solvents, such as water, methanol, and acetone efficiently absorb MW irradiation, whereas non-polar solvents, such as hexane and toluene, do not. MW energy, as a type of non-ionizing radiation, induces ion migration and dipole rotation without altering molecular structures.^46^ Molecules therefore attempt to align with the applied field, generating friction and heat. Upon field removal, thermal agitation restores disorder, thereby releasing energy.^47^ This internal heating is more uniform than that achieved using conventional methods, thereby minimizing temperature gradients in the reaction systems.

MW-assisted reactions demonstrate significant advantages in terms of the reaction speed, efficiency, and selectivity. The effectiveness of a MW-assisted reaction largely depends on the ability of the reaction mixture to absorb MW energy, which is influenced by the choice of solvent and its loss tangent (*δ*), as detailed above. Consequently, solvents can be classified as high, medium, or low MW absorbers, and mixed solvents can be used to tune this property based on the desired reaction system. Furthermore, advancements in MW reactor technologies, particularly silicon carbide reaction vessels and fiber optic temperature probes, have facilitated accurate temperature measurements and a clearer distinction between MW-induced and conventional thermal effects.

Commonly utilized systems include the CEM Discover SP, Biotage Initiator+, Anton Paar Monowave 400, and Milestone Ethos EASY, all of which are designed for safe and efficient chemical synthesis. These reactors can handle reaction scales ranging from a few milligrams to 30–100 grams, depending on the vessel type and system configuration. Method development often involves smaller scales (5–100 mg), while multi-vessel or batch systems facilitate medium scale (0.1–5 g) and larger scale (up to ∼100 g) processes.

### 1.3 Sonochemical synthesis

The application of ultrasonic irradiation represents a new field of study that employs waves with frequencies ranging between 20 kHz and 1 MHz.^48,49^ The use of ultrasound technology in organic synthesis is therefore growing and is particularly crucial in the field of green chemistry. The effects observed under ultrasonic conditions are attributed to cavitation, which is a physical process wherein vaporous and gaseous cavities form, expand, and collapse in the irradiated liquid. High local temperatures and pressures are produced as a result, thereby promoting mass transfer and causing turbulent flow. In organic synthesis, ultrasonication has emerged as a useful method, especially for multicomponent reactions, which render it possible to efficiently synthesize complicated organic molecules from easily accessible starting materials.^50–55^ Indeed, the sonochemical synthesis of heterocycles has gained significant attention among both organic and medicinal chemists worldwide. This technique accelerates reactions by increasing product yields, reducing reaction times, and improving reaction selectivities. Unlike conventional methods, ultrasound synthesis is straightforward, cost-effective, and widely accessible. Additionally, it often eliminates the requirement for external heating, as the ultrasonic waves themselves act as catalysts, ultimately boosting the reaction rate.

### 1.4 Mechanochemical synthesis

Mechanochemical techniques are becoming more widely adopted as greener synthetic methods in response to the need for safer and cleaner chemical processes. While the significance of solvents in the extraction and purification steps remains crucial, mechanochemical techniques have been demonstrated to promote reactant interactions while improving the extent of reaction control and temperature management. The potential value in reducing solvent usage has also been recognized. Ball milling represents a frequently used technique in mechanochemistry, wherein mechanical forces (*e.g.*, compression and friction) promote the reactions, and energy is transferred through impact and shear forces. These conditions enable intimate mixing, increased surface contact, and localized heating, thereby facilitating bond formation and cleavage in the absence of bulk solvents. The regulated, contained conditions provided by modern shaker and planetary mills ensure more consistent results than traditional manual grinding, which is subject to human and environmental variability. Additionally, mechanochemistry can allow reaction pathways to be modified by allowing access to high-energy intermediates and non-classical transition states that would otherwise be inaccessible under conventional conditions. Furthermore, the absence of solvent dielectric effects and temperature control frequently produces distinct reactivity profiles and polymorph results, particularly in co-crystal and supramolecular syntheses.^29^

Commonly utilized equipment includes the Retsch PM100 and PM400 planetary mills, the Retsch MM400 mixing mill, and the Fritsch Pulverisette 7 planetary micro mill, all of which can operate at varied speeds and accommodate different jar sizes (10–500 mL). These mills can handle reaction scales ranging from a few tens of milligrams to several tens of grams, depending on the jar volume and ball-to-powder ratio. The reactions are carried out in jars constructed of stainless steel, zirconia, agate, or tungsten carbide, generally under ambient or inert atmospheres.

### 1.5 Solvothermal synthesis

The solution-based synthetic approach known as solvothermal synthesis entails conducting chemical reactions in a closed system at high pressures and temperatures, usually above the standard boiling point of the solvent. Metal oxides, metal chalcogenides, coordination complexes, metal–organic frameworks, and other functional nanostructures^56,57^ are among some the crystalline materials that are commonly prepared using this technique. Solvothermal circumstances create a particular high-temperature, high-pressure environment that improves the precursor solubility, accelerates the reaction kinetics, and permits regulated nucleation and crystal formation processes. The nature of the solvent, including its polarity, coordinating ability, and dielectric constant, plays a critical role in directing the morphology, size, and phase of the resulting product. Moreover, solvothermal synthesis provides access to metastable phases and complex architectures that are often unattainable under ambient or conventional thermal conditions.^19^

Solvothermal techniques are particularly useful in the preparation of bioactive heterocycles because they are more efficient and selective than traditional synthetic approaches. Furthermore, these methods can reduce energy consumption and lower solvent volumes, rendering them more sustainable and greener options.^58,59^

## 2. N-heterocycles with anticancer activity

Anticancer molecules are compounds that inhibit or destroy cancer cells by targeting specific cellular pathways. They work through various mechanisms, including DNA intercalation, enzyme inhibition, apoptosis induction, and angiogenesis suppression. Common classes include alkaloids, flavonoids, terpenoids, and N-heterocycles, as well as synthetic agents such as tyrosine kinase inhibitors and platinum-based drugs. These molecules form the basis of chemotherapy, immunotherapy and targeted cancer treatments.^60–62^

### 2.1 Microwave-assisted synthesis of anticancer agents

#### 2.1.1 Pyrrole

**Scheme 1:**
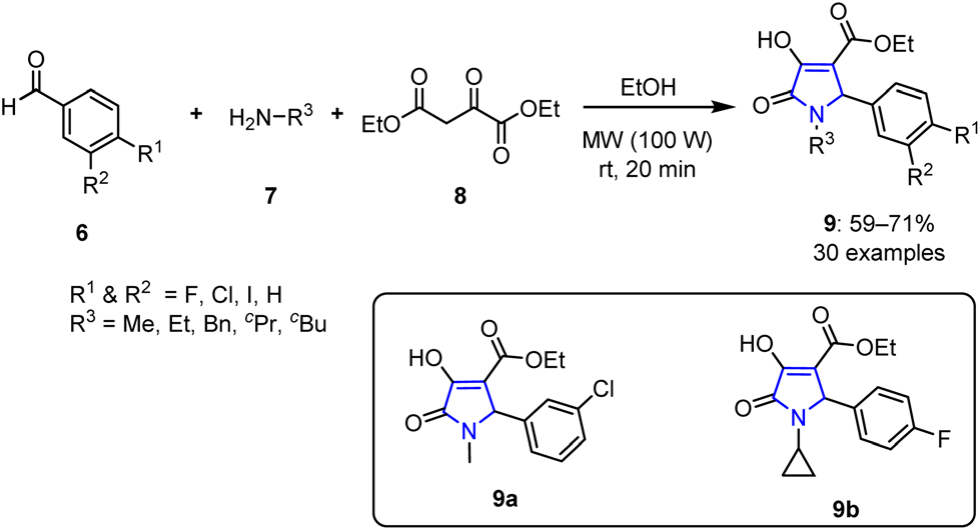
MV-assisted synthesis of various new 3-hydroxy-3-pyrrolin-2-ones 9.

Numerous traditional methods exist for synthesizing pyrroles, including the Knorr, Paal–Knorr, Hantzsch condensation, and Clauson–Kaas reactions. However, when carried out using conventional heating approaches, these methods often demand harsh reaction and work-up conditions, which can negatively affect the product yields. In contrast, newer protocols utilizing MW irradiation offer significant improvements in the efficiency of such reactions. For example, in 2018, Manta *et al.* carried out a multicomponent reaction using equimolecular amounts of aromatic aldehydes 6, primary amines 7, and diethyl oxalacetate 8 in ethanol under 100 WMW irradiation for 20 min to yield various new 3-hydroxy-3-pyrrolin-2-ones 9 in moderate yields 59–71% (Scheme 1).^63^

Among the various synthesized compounds, 9a demonstrated the most promising activity against leukemia cells. Additionally, pyrrole derivatives bearing cyclobutyl substituents exhibited increased efficacies against cervical carcinoma and leukemia, particularly when paired with halogen atoms such as chlorine or iodine (*cf.*, fluorine). *para*-Substitution with a chlorine atom further enhanced the activities of these compounds, particularly in instances of double aromatic substitution. Moreover, the newly synthesized pyrrole-based compound 9b displayed antiviral effects against the yellow fever virus.

#### 2.1.2 Piperidine

**Scheme 2:**
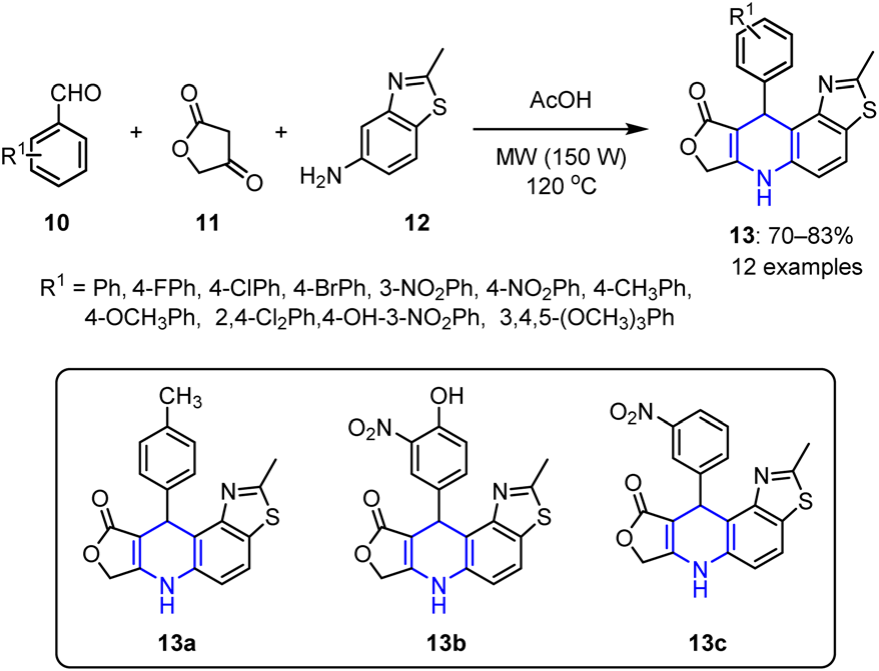
Synthetic protocol for the preparation of 4-aza-podophyllotoxin analogs 13.

The MW-assisted synthesis of piperidines offers faster reactions, higher yields, and improved selectivities under milder reaction conditions. In this context, Shi *et al.* prepared 4-aza-podophyllotoxin analogues 13 in good yields 70–83% *via* a three-component process under MW irradiation (at 150 W). These reactions were performed using various substituted aromatic aldehydes 10 in combination with tetronic acid 11 and benzothiazole 12 (Scheme 2).^64^

**Scheme 3:**
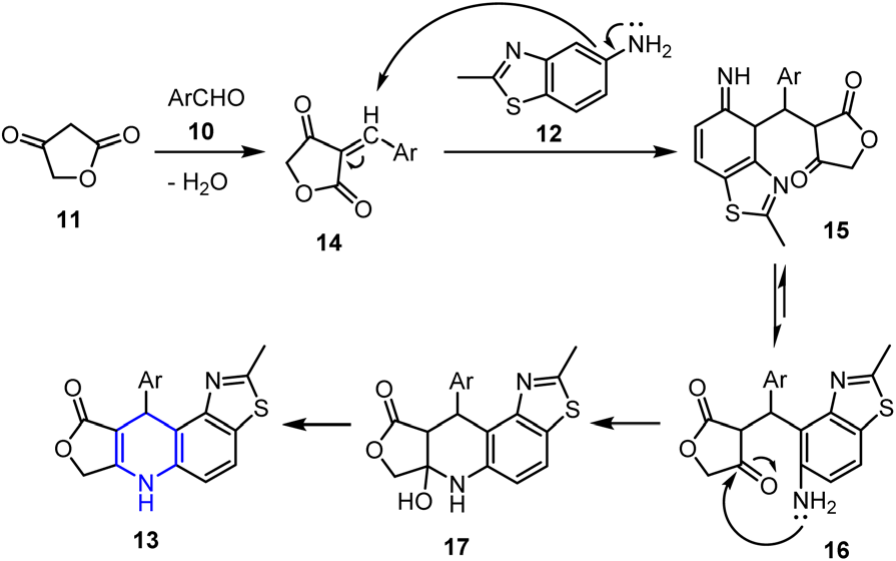
Proposed mechanism for the synthesis of 4-aza-podophyllotoxin analogs 13.

The proposed mechanism for the above transformation (see Scheme 3) begins with a Knoevenagel condensation between aldehyde 10 and tetronic acid 11, forming a β,γ-unsaturated intermediate 14. This is followed by the Michael addition of 2-methylbenzo[*d*]thiazol-5-amine 12 to intermediate 14, yielding an open-chain intermediate 16*via* addition product 15. Finally, intramolecular cyclization and dehydration from 17 produce the final N-heterocyclic product 13.

All synthesized 4-aza-podophyllotoxin analogues 13 demonstrated good cytotoxicities against different cell lines. Specifically, compound 13a, which possesses a –CH_3_ substituent at the *para*-position of the aromatic ring, exhibited the highest cytotoxicity against the MCF-7 breast cancer cell line, with an IC_50_ value of 25.68 μg mL^−1^. Conversely, compound 13b, bearing nitro and hydroxyl substituents, showed the strongest cytotoxic effects across three cell lines, namely the MCF-7 breast cancer cell line (IC_50_ = 56 μg mL^−1^), the M14 malignant melanoma cell line (IC_50_ = 33.9 μg mL^−1^), and the SW1116 colon carcinoma cell line (IC_50_ = 69.4 μg mL^−1^). Additionally, compound 13c, bearing a nitro substituent, demonstrated a significant inhibitory effect against SW1116 colon carcinoma cells, with an IC_50_ value of 25.98 μg mL^−1^. Notably, both compounds 13b and 13c contain *meta*-nitro groups on their aromatic rings, suggesting that this functionality may enhance their cytotoxic effects.

#### 2.1.3 Indole

**Scheme 4:**
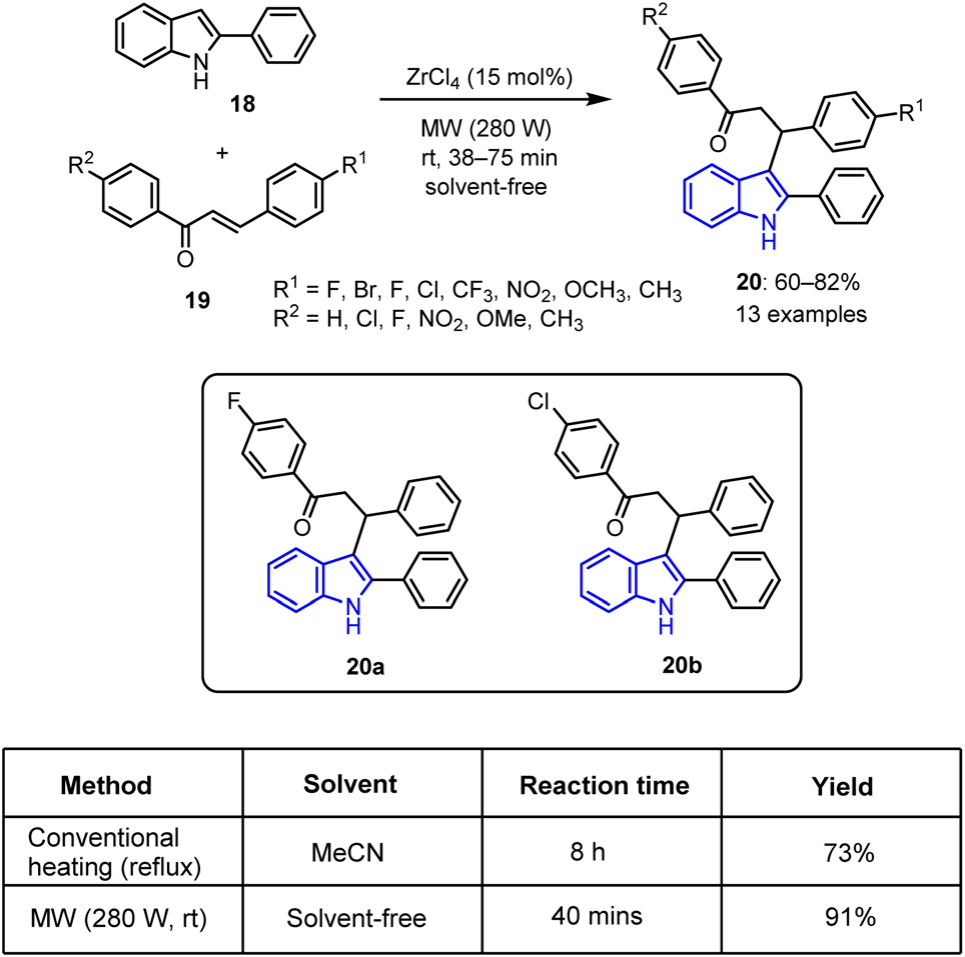
Synthesis of 3-(3-oxoaryl) indole derivatives 20.

Indole is a key heterocyclic scaffold with a versatile reactivity and strong biological relevance. Its structure enables facile functionalization, rendering it of particular importance in drug design, with its derivatives showing diverse pharmacological activities. In this context, Tarun *et al.* detailed the synthesis of 3-(3-oxoaryl) indole derivatives 20 ^65^*via* a Michael addition reaction, wherein 2-phenylindole 18 reacts with a range of chalcones 19. This approach employs ZrCl_4_ as a relatively non-toxic catalyst and is carried out under MW-promoted solvent-free conditions. The reaction yields ranged from 60% to 82%, as detailed in Scheme 4. Notably, the use of MW irradiation produced an efficient reaction system for accomplishing quick and effective chemical transformations with excellent selectivities and yields.

Among the synthesized indole derivatives, compounds 20a and 20b demonstrated effectiveness in inhibiting the growth of the murine melanoma B16F10 cancer cell line along with the MCF-7 human breast cancer cell line, exhibiting maximum IC_50_ values in the range of 10–15 μM. Furthermore, molecular docking studies revealed that compounds 20a and 20b exhibit high docking affinities with the colchicine binding site of the tubulin receptor (10.4 and 9.9 kcal mol^−1^, respectively). These results demonstrate the exceptional potential of compounds 20a and 20b for use as anticancer drugs to inhibit tubulin *via* contact with the colchicine binding site.

#### 2.1.4 Pyrazole

**Scheme 5:**
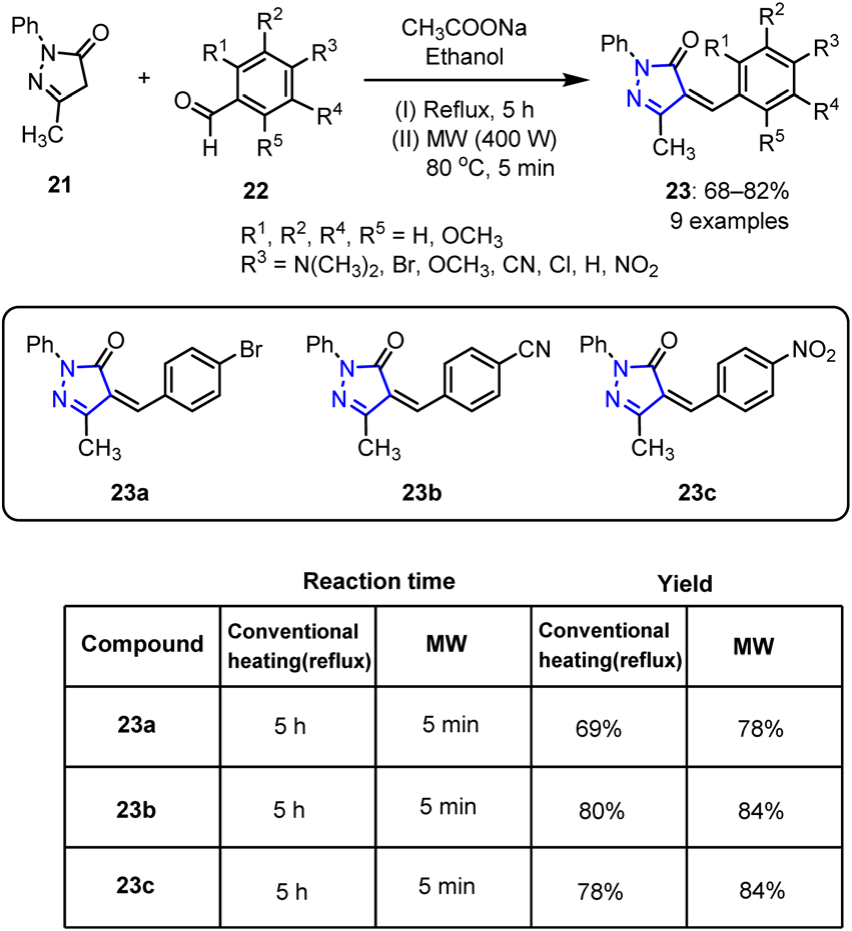
Synthetic route to functionalized 4-arylidene-3-methyl-1-phenyl-1*H*-pyrazol-5(4*H*)-one derivatives 23.

The MW-assisted synthesis of pyrazoles enables rapid, high-yielding reactions with improved purities under mild and eco-friendly conditions. Common routes to pyrazoles include the cyclocondensation of hydrazines with 1,3-dicarbonyl compounds or enones, thereby rendering MW irradiation a valuable tool for efficient pyrazole synthesis in medicinal chemistry. In this context, Shroff *et al.* reported the synthesis of pyrazole derivatives through a novel method,^66^ wherein 3-methyl-1-phenyl-1*H*-pyrazol-5(4*H*)-one 21 was reacted with various substituted aryl aldehydes 22 in the presence of anhydrous sodium acetate and ethanol. MW irradiation was subsequently performed at 400 W and 80 °C for 5 min to produce the desired arylidene derivatives 23 in moderate yields (68–82%), as depicted in Scheme 5.

Among the synthesized arylidene derivatives, compounds 23a, 23b, and 23c demonstrated significant anti-proliferative effects against various cancer cell lines, with IC_50_ values ranging from 2 to 7 μM. Notably, compound 23c proved to be the most potent anticancer compound. Although this compound had no effect on tubulin polymerization, it effectively induced apoptosis in drug-resistant cancer cells by increasing the expression of mitochondria-dependent pro-apoptotic markers, namely Bax and caspase-3. This compound therefore demonstrates an encouraging anticancer activity, wherein it induces cancer cell death by interacting with the mitochondria.

#### 2.1.5 Thiadiazole

**Scheme 6:**
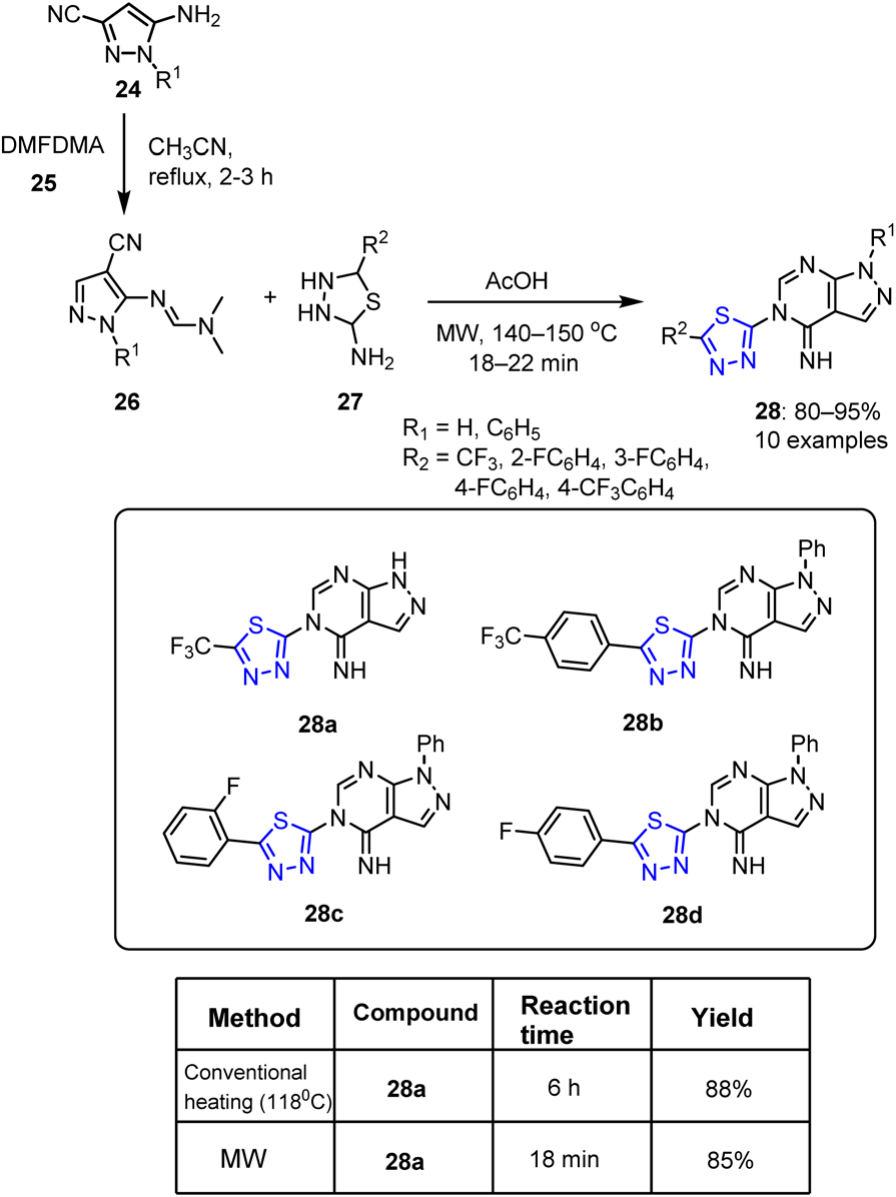
Synthetic protocol to functionalized pyrazolo pyrimidines 28 bearing 1,3,4-thiadiazole moieties.

Song *et al.* reported the reaction of amino pyrazoles 24 and *N*,*N*-dimethyl formamide dimethyl acetal (DMFDMA) 25 in the presence of acetonitrile to yield the corresponding amidines 26.^67^ Subsequent cyclocondensation with suitable substituted-1,3,4-thiadiazole derivatives 27 in acetic acid under MW irradiation yielded a novel series of pyrazolo pyrimidine derivatives 28 incorporating 1,3,4-thiadiazole moieties in yields of 80–95% (Scheme 6).

It was demonstrated that when a hydrogen atom (*cf.*, a phenyl substituent) occupies the R^1^ position of the pyrazole ring, these compounds exhibited significantly greater anticancer activities. Additionally, compounds bearing trifluoromethyl 28a, 4-trifluoromethyl benzene 28b, 2-fluorobenzene 28c, and 4-fluorobenzene 28d substituents also exhibited strong anticancer activities against human leukemia cells (HL-60). Specifically, compounds 28a and 28b were found to be more effective than doxorubicin. Notably, the addition of a CF_3_ group led to a significantly enhanced biological activity.

#### 2.1.6 Imidazole

**Scheme 7:**
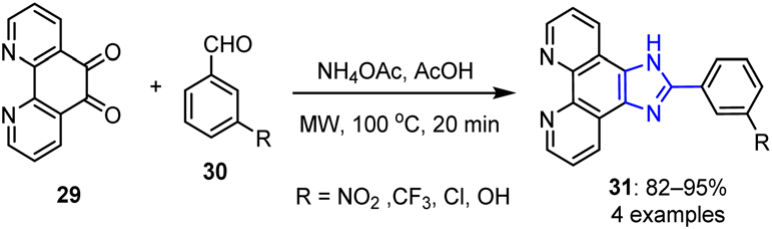
Synthesis of imidazo[4,5-*f*]-1,10-phenanthroline derivatives 31 under MW irradiation.

Conventional routes to imidazole typically require prolonged heating, strong acids, or dehydrating agents, and often result in moderate yields along with side product formation. MW-assisted synthesis overcomes these limitations by enabling rapid, uniform heating, thereby reducing reaction times, improving yields and selectivities, and allowing solvent-free or greener conditions to be employed. Imidazoles are efficiently synthesized *via* the Debus–Radziszewski condensation of a 1,2-dicarbonyl compound and an aldehyde in the presence of ammonium acetate. This simple, versatile method often employs ethanol or acetic acid, and can be accelerated by MW irradiation to give higher yields and shorter reaction times. In 2017, Zheng *et al.* investigated the reactions between phendione 29 and various aromatic aldehydes 30 in the presence of glacial acetic acid and ammonium acetate under MW irradiation.^68^ As a result, various phenanthroimidazole derivatives 31 were obtained in excellent yields of 82–95% (Scheme 7).

The synthesized phenanthroimidazole derivatives 31 were found to effectively inhibit the formation and proliferation of human hepatocarcinoma SMMC7721 cells, human lung adenocarcinoma A549 cells, and human colorectal carcinoma SW620 cells. The maximum inhibitory activity of 31 was an IC_50_ 15.03 μM, with a reduced toxicity being observed against normal cells. Further research showed that the nitro-substituted compound could disperse in the mitochondria of A549 cells, triggering cell death by G1 phase arrest. Furthermore, it showed a moderate affinity for binding to the Bcl-2 G-quadruplex DNA. These compounds also showed potential inhibitory effects against tumor cell development.

#### 2.1.7 Oxadiazole

**Scheme 8:**
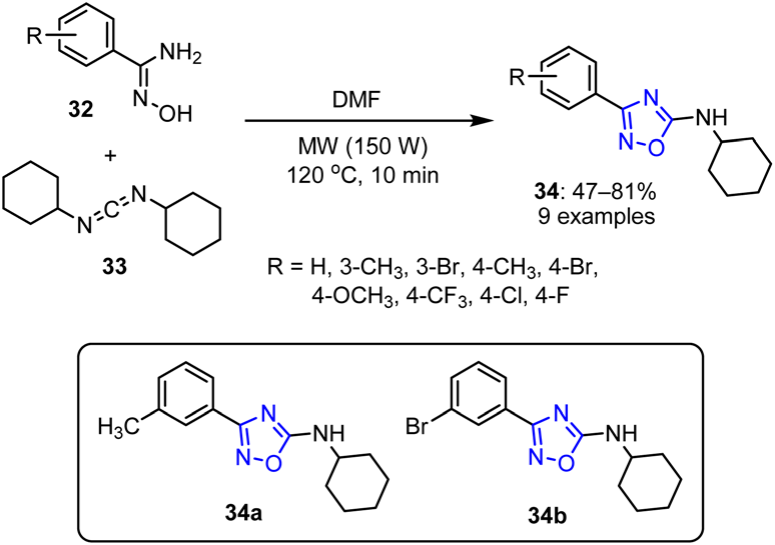
Synthesis of *N*-cyclohexyl-1,2,4-oxadiazole derivatives 34.

In 2018, a study by Oliveira *et al.* investigated the reaction of aryl amidoximes 32 with *N*,*N*-dicyclohexylcarbodiimide (DCC) 33 under MW irradiation at 150 W in DMF.^69^ This methodology afforded 1,2,4-oxadiazole derivatives 34 in moderate-to-good efficiencies, achieving yields between 47% and 81% (Scheme 8).

**Scheme 9:**
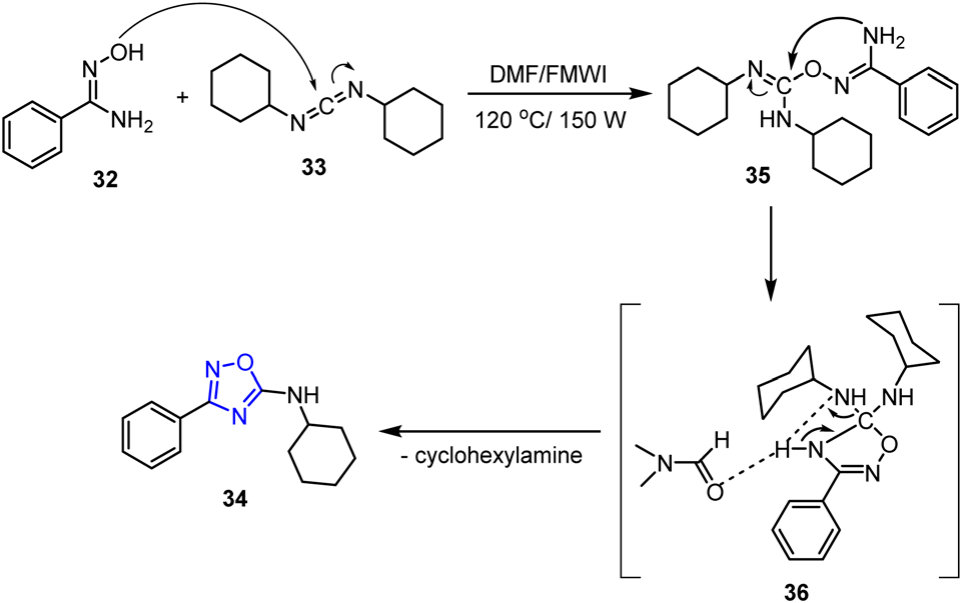
Plausible reaction pathway for the synthesis of 1,2,4-oxadiazole derivatives 34.

The proposed reaction mechanism begins with the nucleophilic attack of benzamidoxime 32 on DCC, forming intermediate 35. The reaction proceeds through a transition state 36, and is accelerated by DMF due to its high dielectric constant, which renders this solvent more reactive under MW irradiation. Solvent-facilitated proton transfer then leads to the elimination of cyclohexylamine, yielding the final product 34 (Scheme 9).

The cytotoxic activities of the samples were subsequently evaluated using the MTT assay. Specifically, they were tested at concentrations of 100 μM for HCT-116 cells and 25 μM for the other cell lines. After dilution in the culture media, the compounds were added to each well, and the MTT assay was employed to determine the IC_50_ values of the active compounds against the B16F10, SNB-19, PC-3, HCT-116, and L929 cell lines after incubation for 72 h. Among the synthesized compounds, *meta*-substituted compounds 34a and 34b showed the most promising anticancer effects, with IC_50_ values of 13.62 mM against SNB-19 and 21.74 mM against PC-3.

#### 2.1.8 Quinolines

**Scheme 10:**
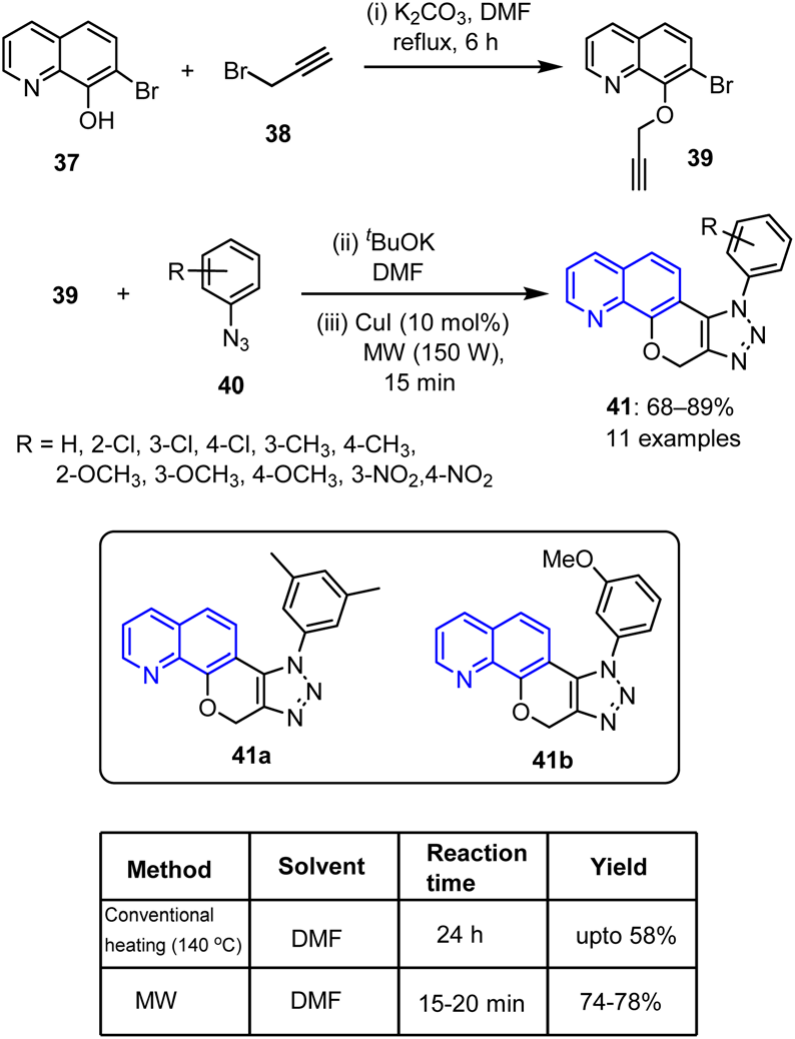
Synthetic route to fused triazolyl pyranoquinolines 41.

Traditional synthetic approaches to quinoline, such as the Skraup and Friedländer reactions, often require strong acids, high temperatures, and extended durations, resulting in safety issues and limited efficiencies. MW-assisted synthesis offers a technically superior alternative by providing rapid thermal activation, enhanced yields, reduced reaction times, and improved functional group tolerances under milder and often solvent-free conditions. In this context, Babu *et al.* prepared emergent fused triazolyl pyranoquinolines 41*via* a three-stage process (Scheme 10).^70^ Initially, 7-bromoquinolin-8-ol 37 was treated with K_2_CO_3_ in DMF, followed by the addition of propargyl bromide 38. After heating the resulting mixture under reflux for 6 h, 7-bromo-8-(prop-2-ynyloxy)quinoline 39 was obtained. Subsequently, intermediate 39 was treated with aryl azide 40, ^*t*^BuOK, and DMF, prior to a subsequent reaction with copper iodide under MW irradiation to produce [1,2,3]triazolo-pyrano[3,2-*h*] quinolines 41 in yields of 68–89%.

The cytotoxic activities of the synthesized compounds were subsequently tested against the alveolar carcinoma (A-549), breast carcinoma (MCF-7), and cervical carcinoma (HeLa) cell lines. Compound 41a, bearing a dimethyl phenyl substituent, showed a strong activity against HeLa and MCF-7 cells, with IC_50_ values of 11.42 ± 0.6 and 16.88 ± 0.5 μM, respectively. Additionally, compound 41b, bearing a benzyloxy substituent on its triazole ring showed a moderate activity, with IC_50_ values of 15.67 ± 0.6 and 31.44 ± 0.5 μM against the HeLa and MCF-7 cell lines, respectively. Alkyl and alkoxy substituents on the triazole ring also led to enhanced activities. In contrast, electron-withdrawing substituents, such as chloro and nitro groups, exhibited fair-to-inadequate efficacies against all three cell lines.

#### 2.1.9 Azapurines

**Scheme 11:**
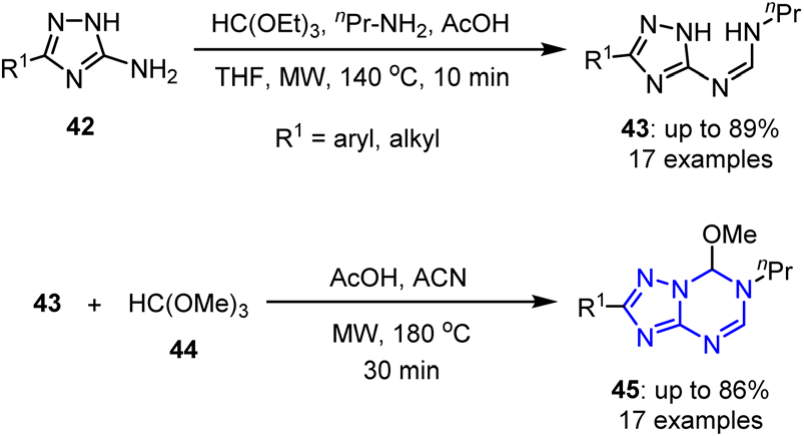
MW-assisted synthetic methodology for the preparation of 6-methoxy-5,6-dihydro-5-azapurines 45.

Alena *et al.* reported the MW-assisted synthesis of novel 6-methoxy-5,6-dihydro-5-azapurines 45, which possess a purine-like framework and demonstrate potential applications in drug discovery.^71^ This rapid and straightforward protocol employs easily accessible reagents, such as trimethyl orthoformate 44, acetic acid, and *N*,*N*-disubstituted formamidines 43 derived from 1,2,4-triazol-5-amines 42.^72–75^ Biological evaluations revealed that certain compounds decreased the viabilities of HepG2 (liver) and A549 (lung) cancer cell lines in a dose-dependent fashion, while showing minimal interactions with five purinergic receptors. These results highlight their selective anticancer activities and underscore their potential as promising candidates for further drug development (Scheme 11).

#### 2.1.10 Oxindole

**Scheme 12:**
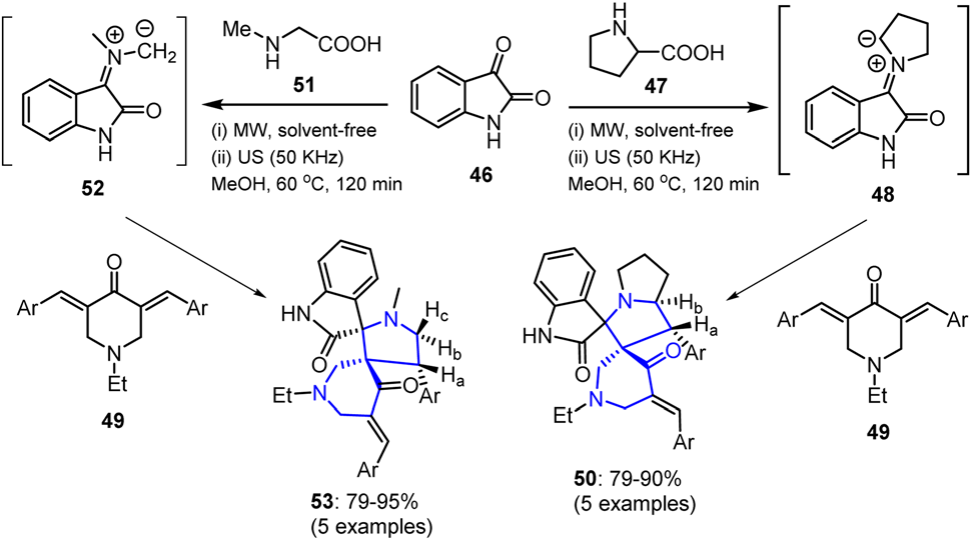
Synthesis of spiropyrrolizine 50 and pyrrolidineoxindole 53 derivatives.

Spirocyclic bioactive N-heterocycles are a unique class of compounds featuring a spiro junction wherein two rings share a single atom. The incorporation of nitrogen into the ring system enhances their biological activities, rendering them valuable scaffolds in the field of drug discovery.^76–78^ In this context, Dina *et al.* synthesized novel spiropyrrolizine 50 and pyrrolidineoxindole 53 derivatives *via* the chemo- and regioselective reaction of bis[arylmethylidene]piperidin-4-ones 49, isatin 46, and either l-proline 47 or sarcosine 51 (Scheme 12).^79^ The reaction was performed under conventional, ultrasonic, and MW-assisted conditions, resulting in efficient product formation through 48 or 52 as an intermediate. The resulting derivatives were evaluated for their antitumor activities against MCF-7 (breast cancer) and HepG2 (liver cancer) cell lines.

### 2.2 Sonochemical synthesis of anticancer agents

#### 2.2.1 Pyrimidine

**Scheme 13:**
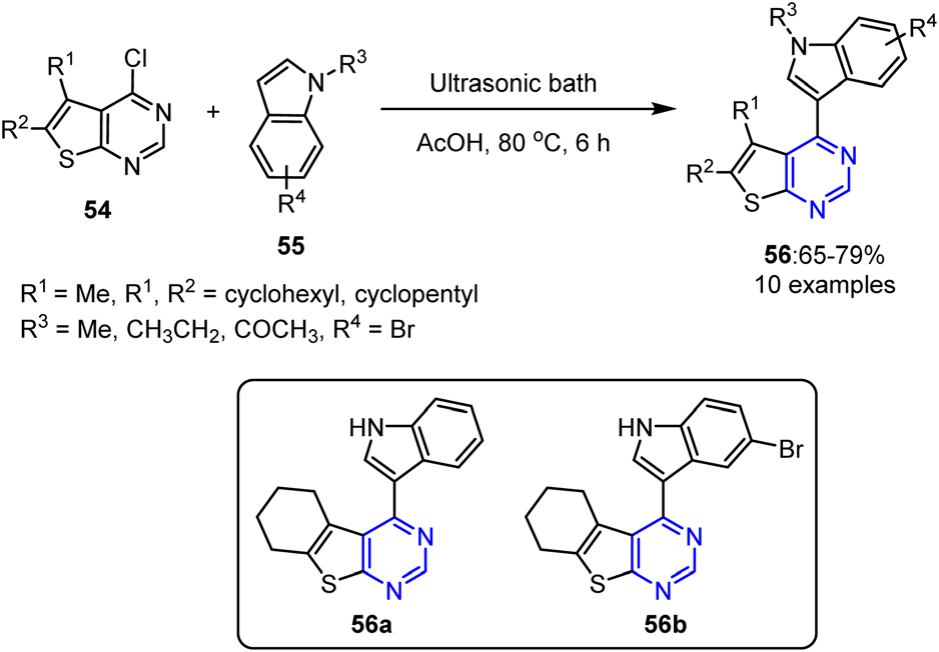
Sonochemical synthesis of 4-(1*H*-indol-3-yl)thieno[2,3-d]pyrimidine derivatives 56.

A sonochemical approach was employed by Sowmy *et al.* to prepare 4-(1*H*-indol-3-yl)thieno[2,3-*d*]pyrimidines 56 through the heteroarylation of 4-chlorothieno[2,3-d]pyrimidines 54 with diverse indoles 55.^80^ The reaction, which was promoted by acetic acid (AcOH) as both a solvent and a catalyst, yielded the desired products in good yields. This metal-free strategy offers benefits such as straightforward conditions, a faster reaction time, and green energy utilization. Nevertheless, the technique failed with *N*-acetyl indole, demonstrating a key limitation (Scheme 13).

**Scheme 14:**
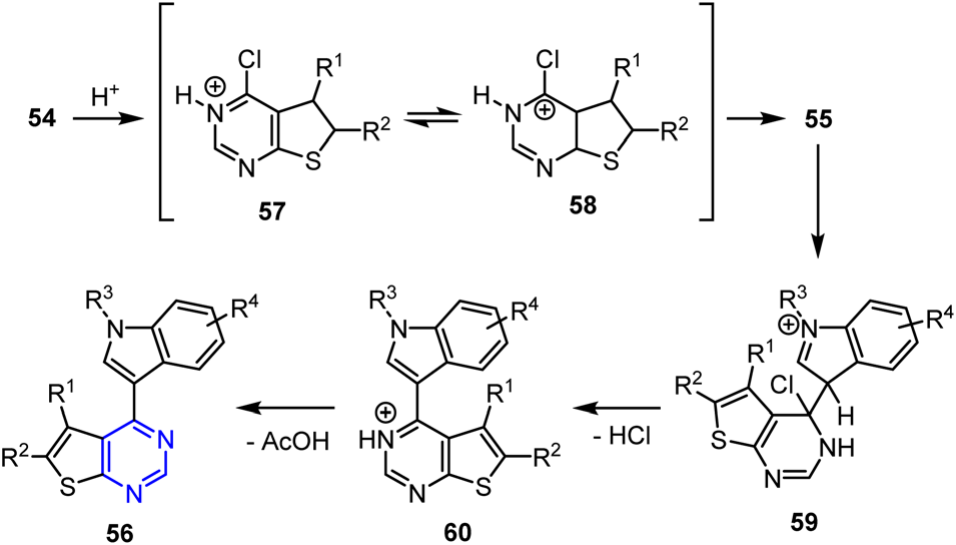
Sonochemical synthesis of 4-(1*H*-indol-3-yl)thieno[2,3-*d*]pyrimidine derivatives 56*via* a heteroarylation reaction in the presence of AcOH.

This reaction began with the ultrasound-promoted protonation of the azomethine nitrogen atom in chlorinated substrate 54, generating resonance-stabilized intermediate 57. The resulting partial positive charge on the chlorine-bonded carbon atom facilitated nucleophilic attack by the C-3 position of indole 55, producing adduct 58. The elimination of HCl *via* intermediates 59 and 60 resulted in the formation of the target 4-(1*H*-indol-3-yl)thieno[2,3-*d*]pyrimidine derivatives 56. This reaction worked efficiently under open air and nitrogen atmospheres, ruling out the involvement of a radical mechanism (Scheme 14).

The newly synthesized thieno[2,3-*d*] pyrimidine derivatives 56 were assessed for their TNF-α inhibition abilities *in vitro* (10 μM), using rolipram and thalidomide as positive controls. Among the tested derivatives, compounds 56a and 56b exhibited 79.3 ± 2.8% and 71.5 ± 1.2% TNF-α inhibition, respectively, compared to 96.3 ± 0.7% for rolipram (at 10 μM) and 63.9 ± 3.9% for thalidomide (200 μM). Consequently, compounds 56a and 56b emerged as viable lead molecules, showing greater TNF-α inhibitory activities than thalidomide despite being less potent than rolipram.

**Scheme 15:**
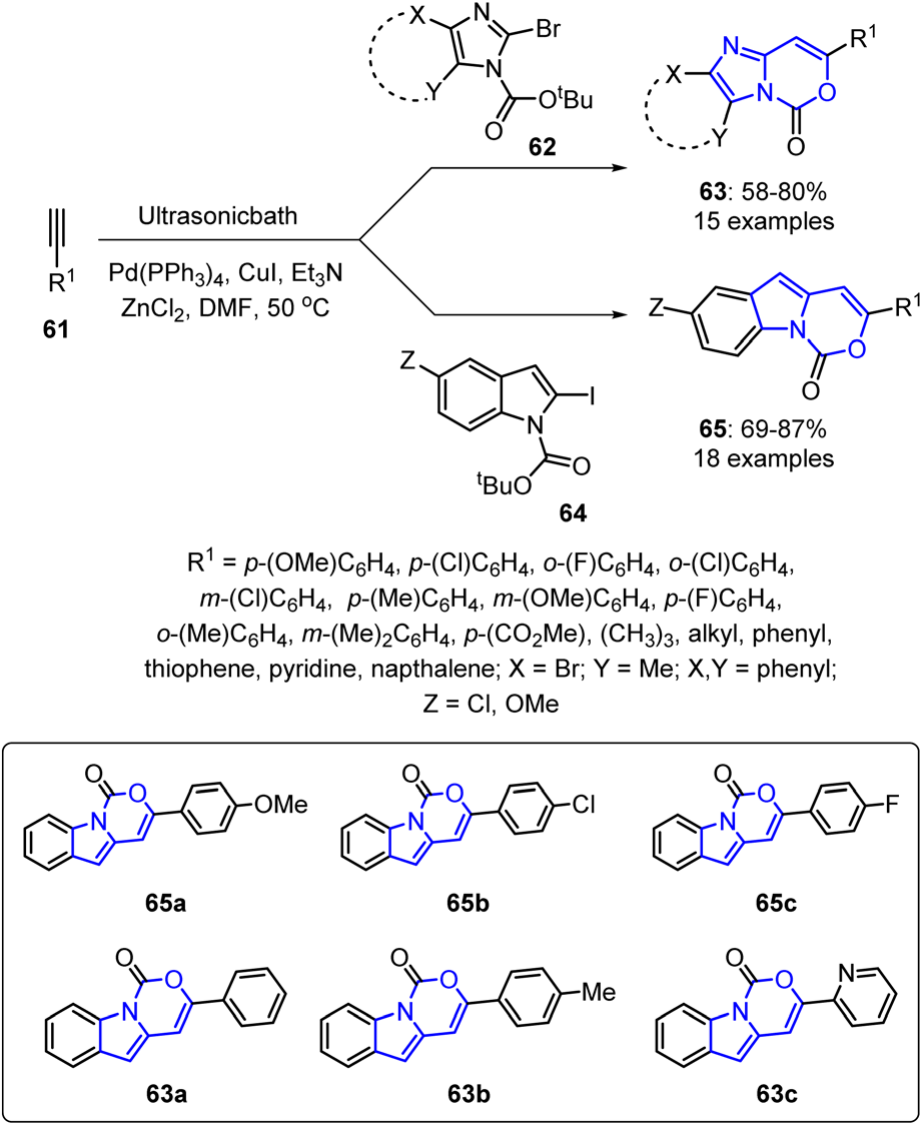
Sonochemical synthesis of oxazinone derivatives 63 and 65.

Additionally, Venkata *et al.* reported a sonochemical approach toward oxazinone derivatives 63 and 65 using Pd/Cu catalysts and ZnCl_2_. This reaction involved coupling 2-iodoindole derivatives 62 and 64 with numerous terminal alkynes 61 in the presence of (PPh_3_)_4_Pd, CuI, and ZnCl_2_ in DMF under ultrasonic irradiation.^81,82^ The reaction proceeded *via* a cascade pathway involving C–C coupling followed by intramolecular cyclization in a regioselective manner, yielding the target compounds in good-to-acceptable yields. This methodology offers advantages such as mild reaction conditions, shorter reaction times, and the use of an eco-friendly energy source (Scheme 15).

Following *in vitro* screening, compounds 65a, 65b, and 65c were identified as preliminary leads for TNF-α inhibition. Furthermore, *in silico* docking studies using the TNF-α protein (PDB 7JRA and 6 × 81) predicted 65a, 63a, 63b, and 63c as potential leads. These compounds interacted with common residues, such as LEU233, TYR135, TYR227, LEU133, ILE231, and LEU196 in 7JRA and TYR59, LEU57, TYR119, and GLY121 in 6 × 81. *In vitro* evaluations confirmed that 63a, 63b, and 63c were the most active compounds. Additionally, structural analysis revealed that compounds bearing the tricyclic fused ring system 1*H*-benzo[4,5]imidazo[1,2-*c*][1,3]oxazin-1-one exhibited superior TNF-α inhibition capabilities than those containing a bicyclic fused ring (5*H*-imidazo[1,2-*c*][1,3]oxazin-5-one). SAR analysis indicated that the C-3 aryl group in the benzoimidazo-1,3-oxazinones followed the activity order: C_6_H_5_ > *p*-MeC_6_H_4_ > 2-pyridyl > *p*-MeOC_6_H_4_ > *p*-ClC_6_H_4_, whereas substitution with a 3-thienyl group at the C-3 position led to a reduced efficacy.

**Scheme 16:**
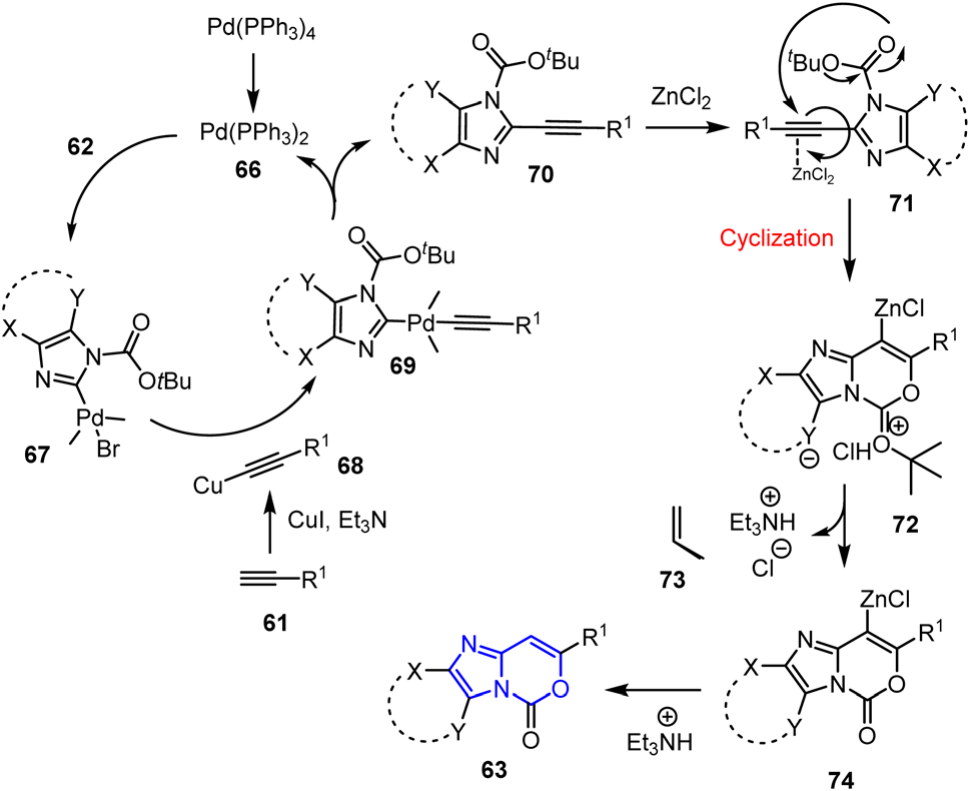
Sonochemical coupling–cyclization mechanism for the synthesis of imidazo-1,3-oxazinones 63.

The reaction mechanism for the formation of products 63 begins with the ultrasound-assisted generation of the active Pd(0) species 66, which undergoes oxidative addition to the halide 62, forming the Pd(ii) species 67. Transmetalation with *in situ* generated Cu-acetylide 68 affords the heteroarene-Pd(ii)-alkynyl complex 69. Sonochemical conditions facilitate the subsequent reductive elimination to yield internal alkyne 70, thereby regenerating the active Pd catalyst. Furthermore, activation of the 70 triple bond by ZnCl_2_ generates 71, which undergoes 6-*endo-dig* cyclization through intramolecular nucleophilic attack by the neighboring –CO_2_^*t*^Bu oxygen atom on the polarized triple bond, generating the cyclic organo-Zn intermediate 72. The ultrasound-assisted release of 2-methylprop-1-ene 73 yields the neutral Zn-species 74, which spontaneously quenches to form the final product 63 (Scheme 16).

### 2.3 Mechanochemical synthesis of anticancer agents

#### 2.3.1 Indole

**Scheme 17:**
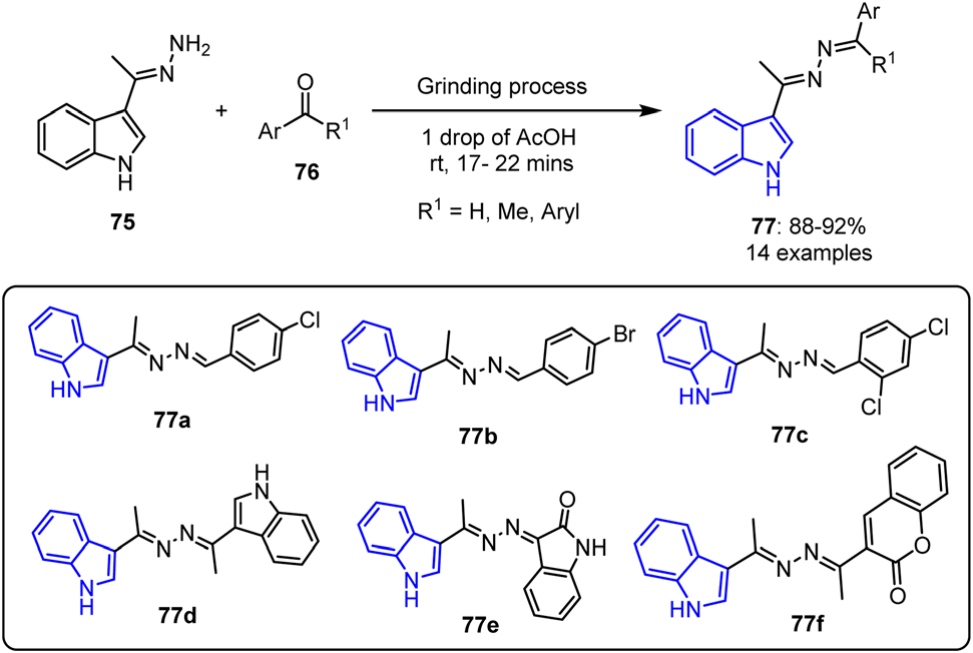
Synthesis of benzaldazine and ketazine derivatives 63 using a grinding methodology.

Ibrahim *et al.* reported an environmentally friendly synthesis of novel benzaldazine and ketazine derivatives 77.^83^ This method involved a solvent-free grinding technique using a catalytic amount of acetic acid to facilitate the condensation of various aldehydes and ketones 76 with 3-(1-hydrazineylideneethyl)-1*H*-indole 75 (Scheme 17). The anticancer potentials of the prepared compounds were evaluated against colon (HCT-116), liver (HepG2), and breast (MCF-7) cell lines using the MTT assay, using doxorubicin as the standard reference drug. With IC_50_ values ranging from 4.27 to 8.15 μM, a number of derivatives (*i.e.*, 77a, 77b, 77c, 77d, and 77e) showed significant efficacies against HCT-116 cells, giving comparable or superior results to doxorubicin (IC_50_ = 5.23 μM). With respect to the HePG-2 cell line, doxorubicin exhibited an IC_50_ value of 4.50 μM, while compounds 77a, 77b, 77c, 77d, 77e, and 77f gave IC_50_ values ranging from 4.09 to 9.05 μM. For the MCF-7 cell line, derivatives 77c, 77d, and 77e demonstrated notable activities (IC_50_ = 6.19–8.39 μM), in contrast to doxorubicin (IC_50_ = 4.17 μM).

#### 2.3.2 Isoxazole

**Scheme 18:**
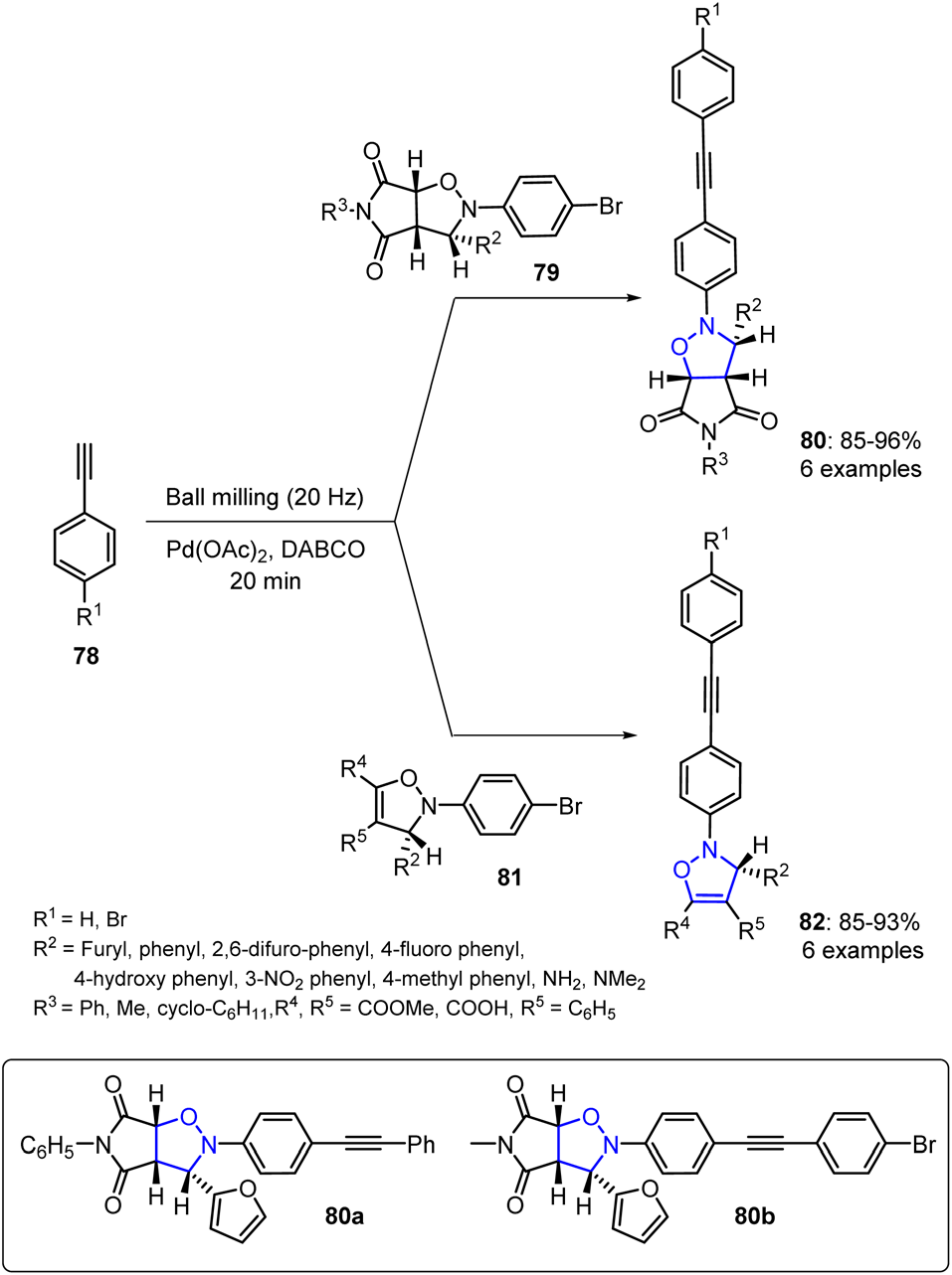
Sonogashira cross-coupling reaction to generate isoxazoline 82 and isoxazolidine 80.

Bhaskar and Esmita described the synthesis of novel heterocyclic derivatives 80 and 82*via* a Sonogashira cross-coupling protocol and a mechanochemical strategy.^84^ This reaction was conducted in a ball mill under solvent-free conditions, emphasizing the efficient and environmentally sustainable nature of their method. The ball-milling process enabled effective coupling by providing the necessary mechanical energy to activate the reactants, eliminating the requirement for conventional heating or toxic solvents (Scheme 18).

Screening of the synthesized compounds indicated that the majority displayed notable cytotoxic effects against prostate, leukemia, breast, and lung cancer cell lines. Among the cycloadducts, isoxazolidine derivatives 80a and 80b exhibited optimal IC_50_ values against the HCT-8, PC-3, and MDA-MB-23 cell lines, demonstrating superior activities than other isoxazolidine and isoxazoline analogues. Based on these results, derivatives 80a and 80b were selected for further cell cycle analysis, which is currently underway.

## 3. N-heterocycles with antibacterial activities

Antibacterial active compounds are substances that inhibit bacterial growth or kill bacteria by targeting essential cellular processes. They function *via* various mechanisms, including cell wall disruption, the inhibition of protein synthesis, interfering with DNA replication, and blocking metabolic pathways.^85,86^

### 3.1 Microwave-assisted synthesis of antibacterial agents

#### 3.1.1 Pyrrole

**Scheme 19:**
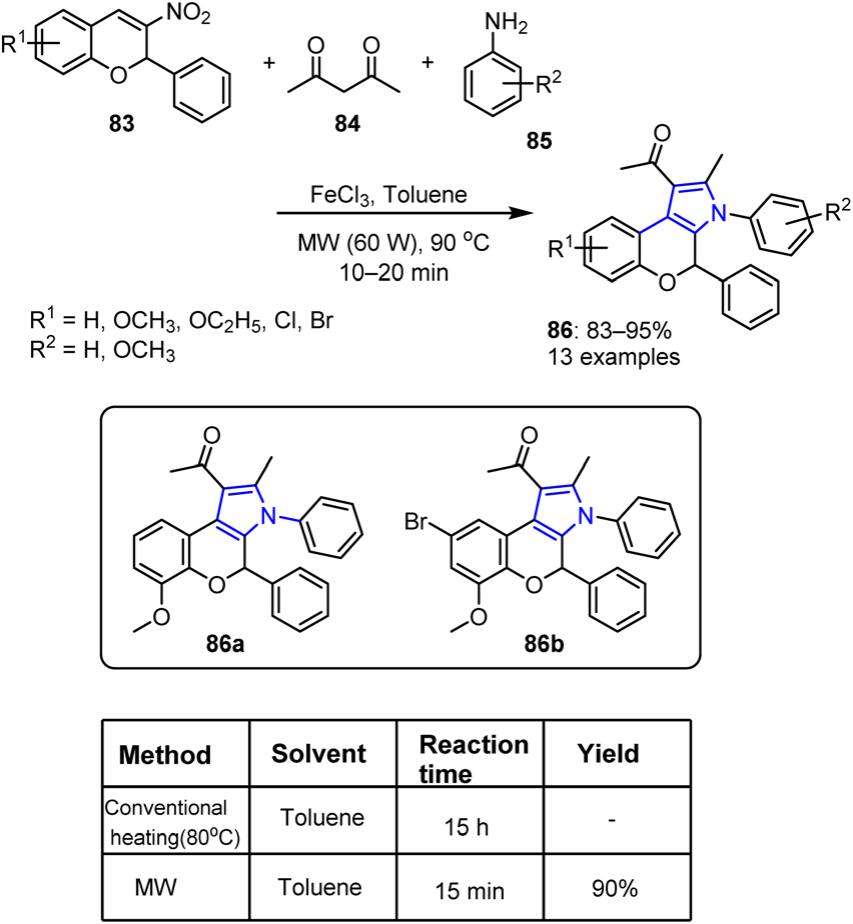
Synthesis of chromene-fused pyrrole derivatives 86 from 3-nitro-2-phenyl-2*H*-chromenes 83.

Baral *et al.* reported the synthesis of novel fused chromene-pyrrole derivatives 86.^87^ In this process, numerous functionalized 2*H*-chromenes 83 were reacted with acetylacetone 84 and aniline 85 in the presence of FeCl_3_ as a catalyst in toluene under 60 W MW irradiation for 15 min. As detailed in Scheme 19, the desired chromene-fused pyrrole derivatives 86 were obtained in good yields.

**Scheme 20:**
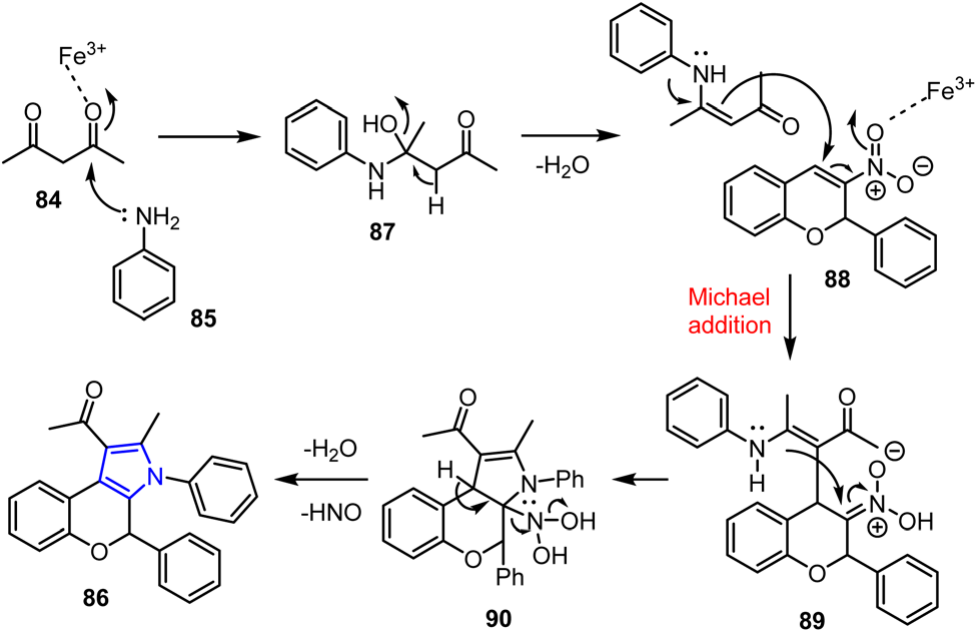
Plausible mechanism for the MW-assisted one-pot synthetic protocol for the preparation of 2*H*-chromene-fused pyrrole derivative 86.

As a Lewis acid, FeCl_3_ catalyzes the multicomponent reaction by activating the carbonyl oxygen atom of the 1,3-dicarbonyl compound. The first step involves the nucleophilic addition of acetylacetone 84 to aniline 85 to generate β-enaminocarbonyl intermediate 87. Subsequently, intermediate 89 is produced *via* a Michael addition reaction with 3-nitro-2*H*-chromene 88, which is catalyzed by FeCl_3_. Compound 89 then undergoes an intramolecular cyclization reaction, resulting in the formation of an intermediate dihydropyrrole 90, which eventually eliminates HNO and water to generate the desired product 86, which possesses a fused heterocyclic structure (Scheme 20).

The produced compounds were evaluated using the agar-well diffusion technique, with gentamicin acting as the reference antimicrobial agent. The assays focused on the Gram-positive bacterium *Staphylococcus aureus* (MTCC7443) and the Gram-negative bacterium *Escherichia coli* (MTCC614), enabling the assessment of the minimum inhibitory concentration (MIC) and the inhibition zone. Among the synthesized compounds, 86a and 86b showed significant activities against *E. coli* and *S. aureus*. Compound 86b exhibited the greatest activity, producing inhibition zones of 18 and 99 mm against *E. coli* and *S. aureus*, respectively, at an MIC of 20 μg mL^−1^. This level of activity is almost comparable to that of gentamicin, which shows inhibition zones of 17 and 19 mm against *E. coli* and *S. aureus*, with an MIC of 20 μg mL^−1^.

#### 3.1.2 Oxadiazole

**Scheme 21:**
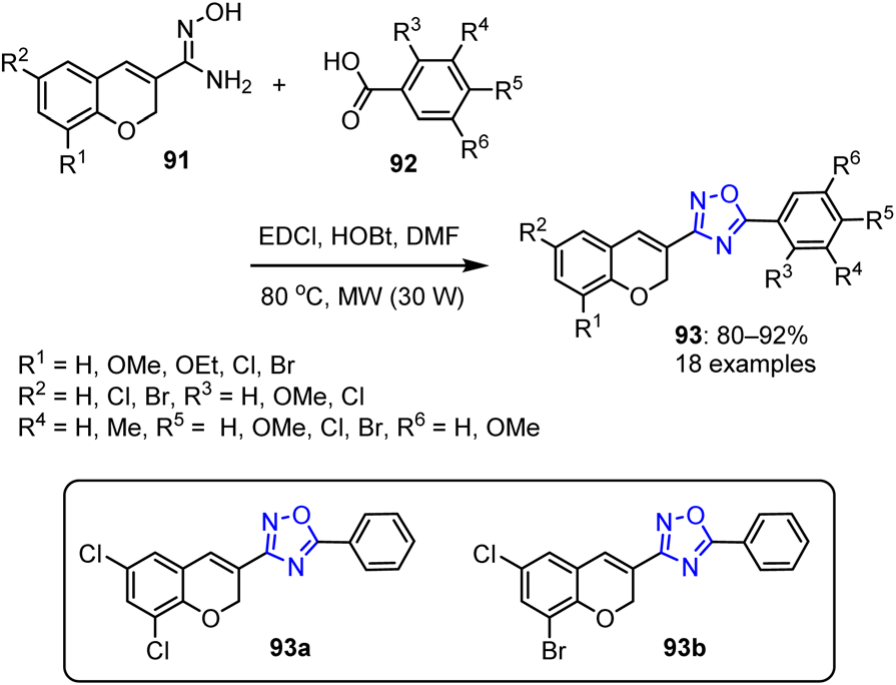
Microwaved-assisted synthetic route for the preparation of fused chromene-oxadiazole derivatives 93.

The same research group synthesized chromene-fused oxadiazole derivatives 93 with yields ranging from 80% to 92%.^88^ This involved the reaction of 2*H*-chromene-3-carboximidamide 91 with various carboxylic acid derivatives 92 in anhydrous DMF. The reaction was facilitated by EDCI/HOBt as the coupling agent and was conducted under MW irradiation (Scheme 21).

Among the produced compounds, 93a and 93b were recognized as the most effective antibacterial agents. These compounds exhibited robust activities against *E. coli* (MTCC614) and *Klebsiella pneumoniae* (MTCC4031). Furthermore, they specifically bound to the DNA gyrase of the targeted bacteria.

#### 3.1.3 Indole

**Scheme 22:**
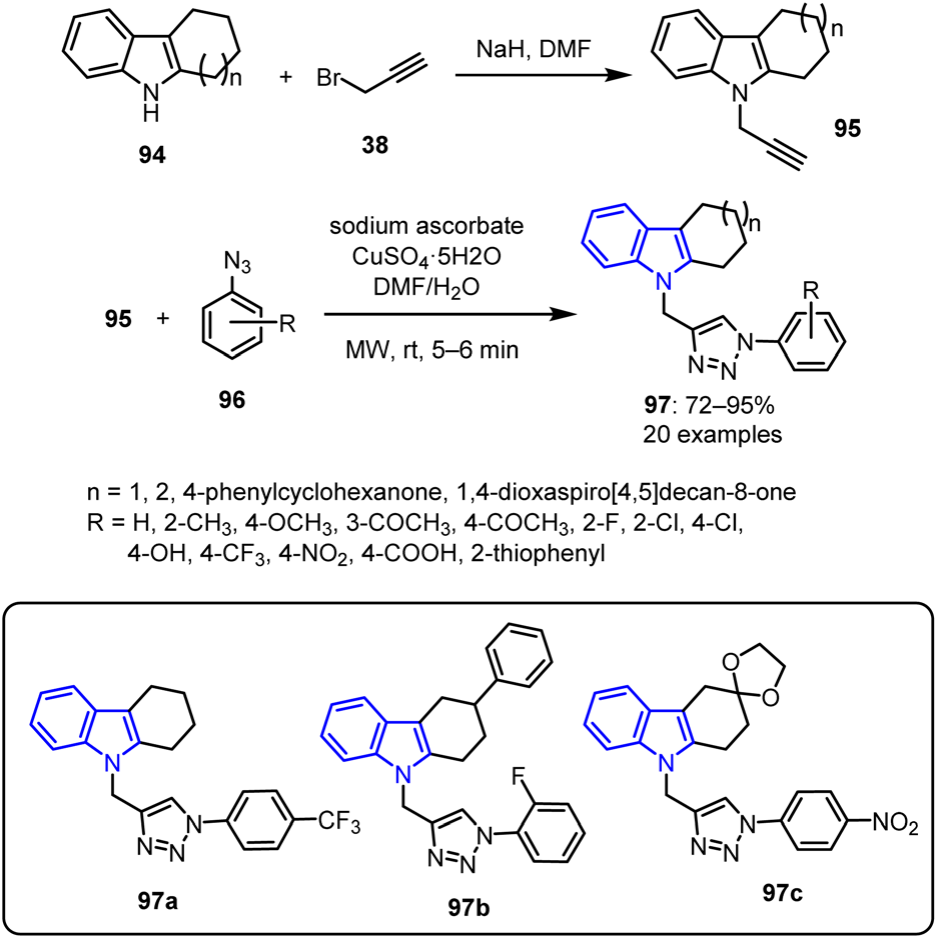
Synthetic pathway for the preparation of novel indole-substituted functionalized 1,2,3-triazole-based carbazole derivatives 95.

Ashok *et al.* outlined a synthetic pathway for the synthesis of functionalized 1,2,3-triazole-based carbazole derivatives 97 bearing indole and triazole as substituents.^89^ Initially, diverse tetrahydrocarbazoles 94 were synthesized by reacting substituted cyclohexanones with phenyl hydrazine.^90^ The resulting tetrahydrocarbazoles 94 then underwent a base-catalysed *N*-alkylation with propargyl bromide 38 in DMF, leading to the formation of *N*-propynyl tetrahydrocarbazoles 95. These alkynes were subsequently reacted with various aromatic azides 94*via* a copper-catalyzed Huisgen [3 + 2] cycloaddition reaction under MW irradiation in the presence of sodium ascorbate and CuSO_4_. This process yielded functionalized derivatives 97 in good yields ranging from 72% to 95% (Scheme 22).

All synthesized compounds were evaluated using well diffusion techniques at concentrations of 10 and 20 μM. Ampicillin served as the reference antibiotic and the inhibition zones were measured in millimeters (mm) and compared with those of the standard drug nystatin. The assays were performed against three Gram-negative bacterial strains as well as two Gram-positive strains. Among the synthesized compounds, those bearing *p*-(trifluoromethyl)phenyl 97a, *o*-fluorophenyl 97b, and *p*-nitrophenyl 97c substituents demonstrated excellent antibacterial efficacies against all tested strains.

#### 3.1.4 Quinoline

**Scheme 23:**
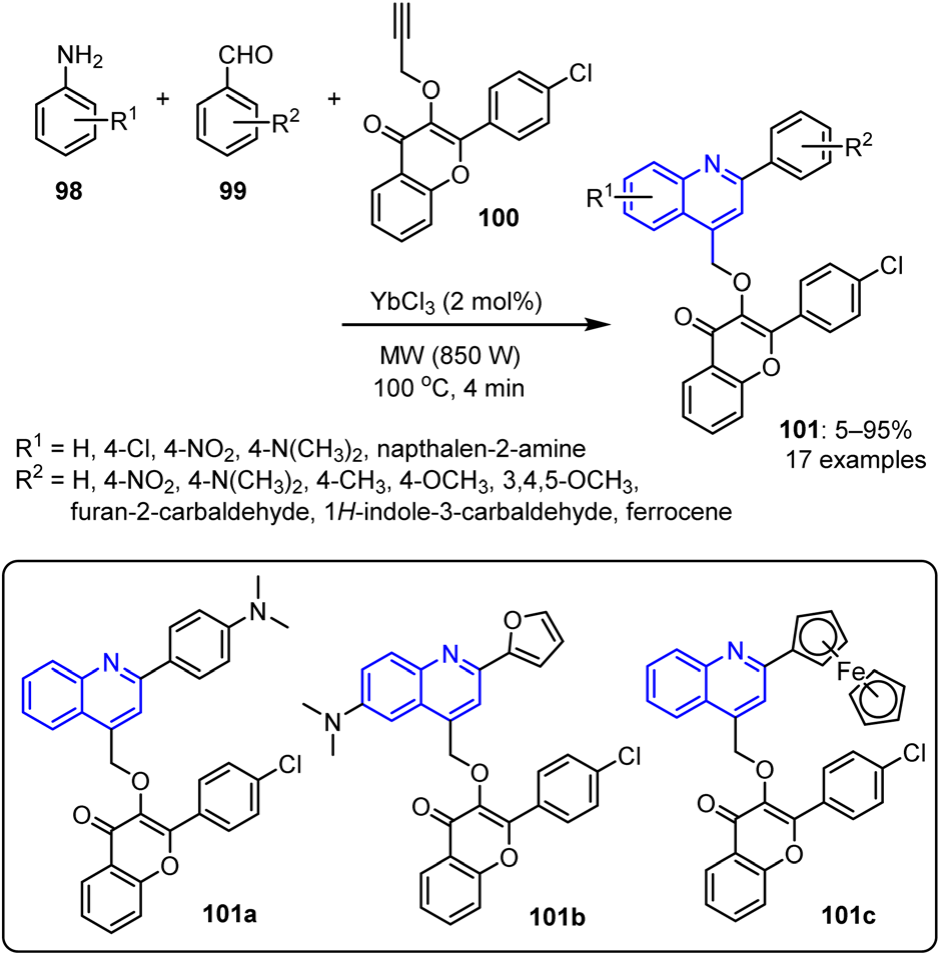
Synthesis of quinolin-4-yl-methoxychromen-4-ones 101.

Sumit *et al.* reported the synthesis of quinolin-4-ylmethoxychromen-4-ones 101.^91^ The reaction sequence involved the coupling of aryl-substituted propynyloxy chromenone 100 with various substituted anilines 98, followed by the addition of substituted aromatic aldehydes 99, under MW-assisted conditions. The process afforded the target compounds 101 with yields varying from 5% to 95% (Scheme 23).

Among the synthesized compounds, those featuring an electron-donating group (*e.g.*, dimethyl amine, 101a) and compounds containing heterocyclic structures (*e.g.*, furan, 101b and ferrocene, 101c) displayed significant antibacterial activities. Specifically, these compounds demonstrated remarkable-to-adequate MICs (0.39–1.56 mg mL^−1^) against *S. aureus*, *E. coli*, and *B. subtilis*.

**Scheme 24:**
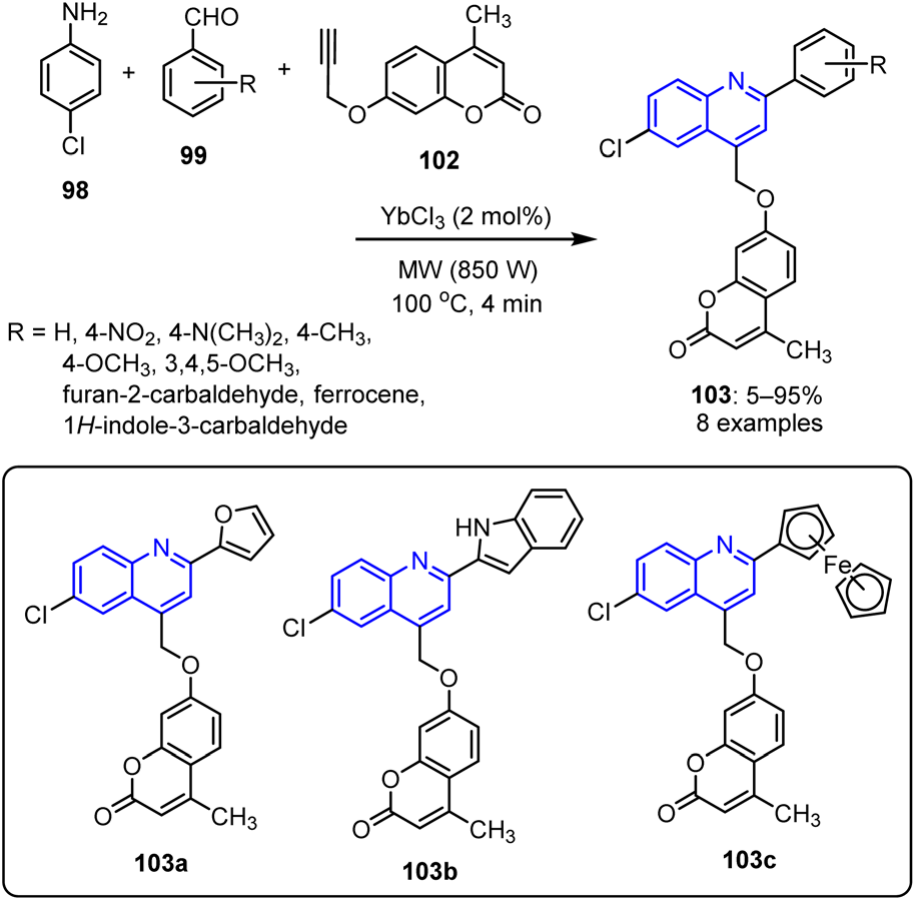
YbCl_3_-catalyzed synthetic protocol for the preparation of arylmethoxy-chromen-2-one derivatives 103.

The same group further synthesized aryloxy chromen-2-ones 103 using a similar synthetic protocol.^91^ This involved the reaction of propynyloxy-2*H*-chromen-2-one 102 with 4-chloro aniline 98 and various substituted aromatic aldehydes 99. This procedure, which was conducted in the presence of YbCl_3_ under MW exposure at 100 °C for 4 min, produced the corresponding quinolin-4-yl methoxy-chromen-2-one derivatives 103 in good yields of up to 94% (Scheme 24). Similar to the quinolin-4-ylmethoxychromen-4-ones 101, the synthesized quinolin-4-yl methoxy-chromen-2-ones bearing heterocyclic substituents (*i.e.*, 103a, 103b, and 103c) exhibited outstanding-to-satisfactory MICs against *S. aureus*, *E. coli*, and *B. subtilis*.

**Scheme 25:**
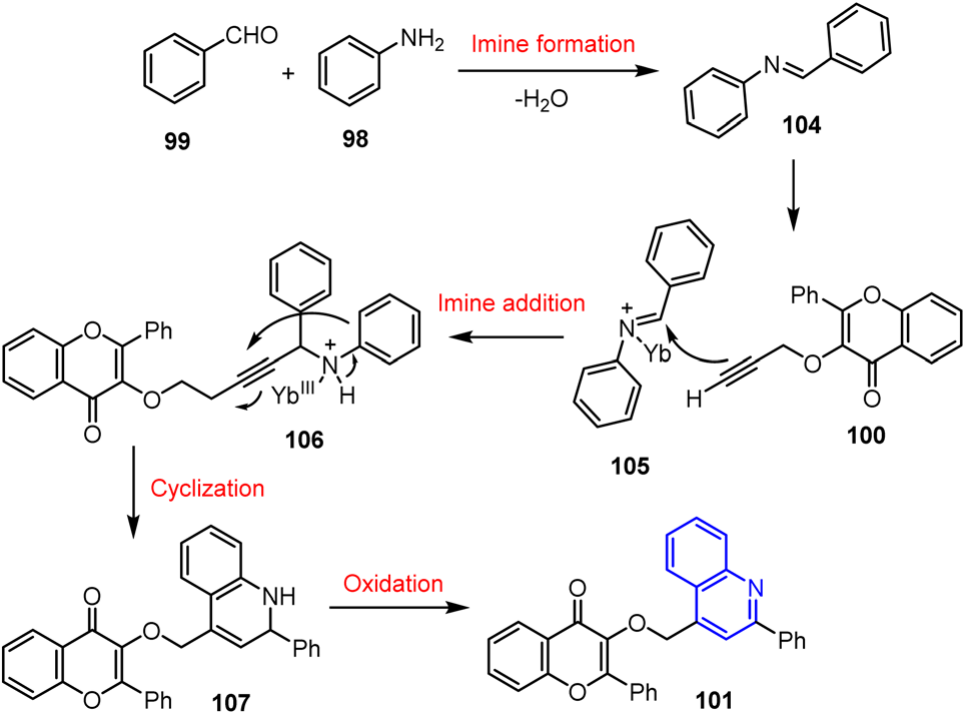
Proposed reaction mechanism for the YbCl_3_-catalyzed one-pot synthesis of quinolin-4-yl methoxy-chromen-4-ones 101.

This one-pot reaction follows a domino pathway, namely imine formation, addition, cyclization, and oxidation pathways, which were catalysed by YbCl_3_. Yb^3+^, which is a hard Lewis acid, coordinates with the imine nitrogen of 104, enhancing its electrophilicity and promoting nucleophilic attack. Its small ionic radius and water tolerance boost its efficiency, facilitating cyclization and oxidation to give a high selectivity and yield (Scheme 25).

**Scheme 26:**
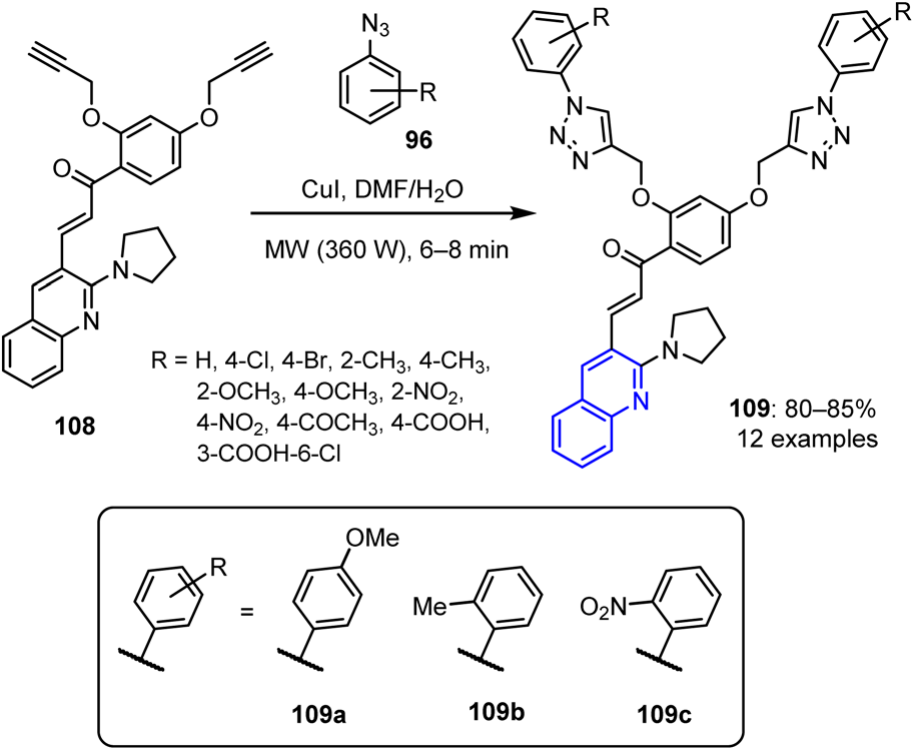
Synthesis of pyrrolidinyl-quinoline- and 1,2,3-triazole-based chalcone derivatives 109.

Pradeep *et al.* reported the synthesis of triazole-based derivatives *via* a [3 + 2] cycloaddition reaction.^92^ This involved the reaction of 2,4-dipropynyloxy-α-substituted acetophenone 108 with aryl azides 96 under MW irradiation at 360 W (Scheme 26). The resulting products, namely pyrrolidinyl-quinoline chalcone hybrid bis-1,2,3-triazoles 109, were obtained in yields of up to 84% after only 6–8 min of irradiation. The starting material, dipropynyloxy-α-substituted acetophenone 108, was also synthesized using MW irradiation. Specifically, this was performed by reacting a methanolic solution of 2-pyrrolidinyl-3-quinoline carbaldehyde and 1-(2,4-bis(prop-2-yn-1-yloxy)phenyl)ethanone in the presence of KOH.^92^

Among the synthesized compounds, the compounds bearing 4-methoxyphenyl 109a, 2-methylphenyl 109b, and 2-nitrophenyl 109c substitutions exhibited strong antibacterial activities. Specifically, they were effective against four bacterial strains, namely *Enterococcus faecalis*, *K. pneumoniae*, *S. aureus*, and *E. coli*. Ampicillin was employed as the reference standard.

#### 3.1.5 Pyrazole

**Scheme 27:**
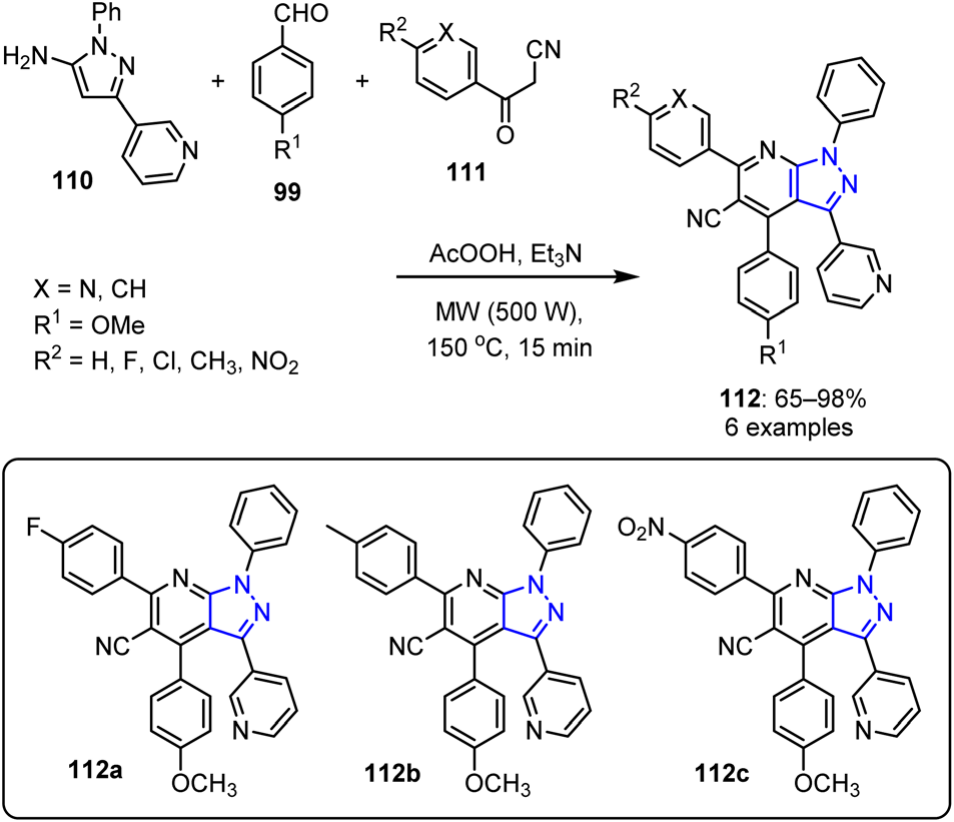
MV-assisted synthesis of 1-aza indazole derivatives 112.

El-Borai *et al.* reported a novel one-pot, multicomponent approach for the synthesis of pyrazolo[3,4-*b*]pyridine derivatives 112 under MW-assisted conditions.^93^ In an acetic acid medium, 5-amino-1-aryl pyrazolylpyridine 110 was reacted with 4-anisaldehyde 99 in the presence of triethylamine. The reaction was performed at 150 °C for 15 min, affording the desired pyrazolo[3,4-*b*]pyridine derivatives 110 in yields of up to 98% (Scheme 27).

The newly synthesized 1-aza indazole derivatives 112a and 112b exhibited moderate antibacterial activities against both Gram-negative bacteria (*Enterobacter cloacae*, *E. coli*, and *Serratia*) and Gram-positive bacteria. Additionally, compound 112c exhibited antifungal properties against *Fusarium oxysporum* and *Penicillium expansum*.

#### 3.1.6 Imidazole

**Scheme 28:**
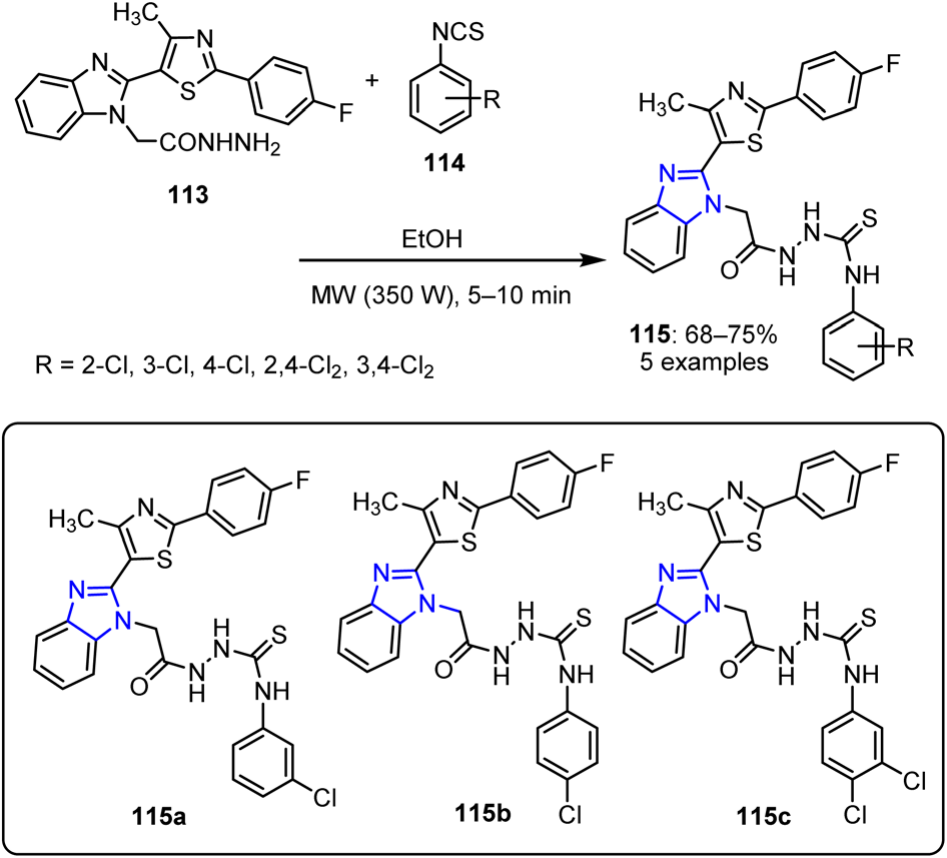
Synthesis of thiosemicarbazide-based imidazole derivatives 115.

Due to its electron-rich nature and ability to participate in hydrogen bonding and metal coordination, imidazole serves as a crucial scaffold in pharmaceuticals, catalysis, and bioinorganic chemistry. The C-2 and C-4/C-5 positions are particularly reactive, enabling diverse functionalization for advanced synthetic applications. In this context, Darekar *et al.* reported a novel synthetic method for creating thiosemicarbazide-based imidazole derivatives 115, as shown in (Scheme 28).^94^ Specifically, they synthesized acetohydrazide-substituted heterocycles 113 through a series of reactions. These compounds were subsequently treated with aryl isothiocyanates 114 under MW irradiation at 350 W for 5–10 min, resulting in the formation of thiosemicarbazide-based imidazole derivatives 115.

The compounds bearing substituents at the *meta* and *para* positions (115a, 115b, and 115c) exhibited significant activities toward Gram-positive and Gram-negative bacterial strains. The bacterial strains selected for evaluation included *Bacillus subtilis* (NCIM 2063), *E. coli* (NCIM 2810), *S. aureus* (NCIM 2079), and *Salmonella abony* (NCIM 2257).

#### 3.1.7 Triazoles and thiadiazoles

**Scheme 29:**
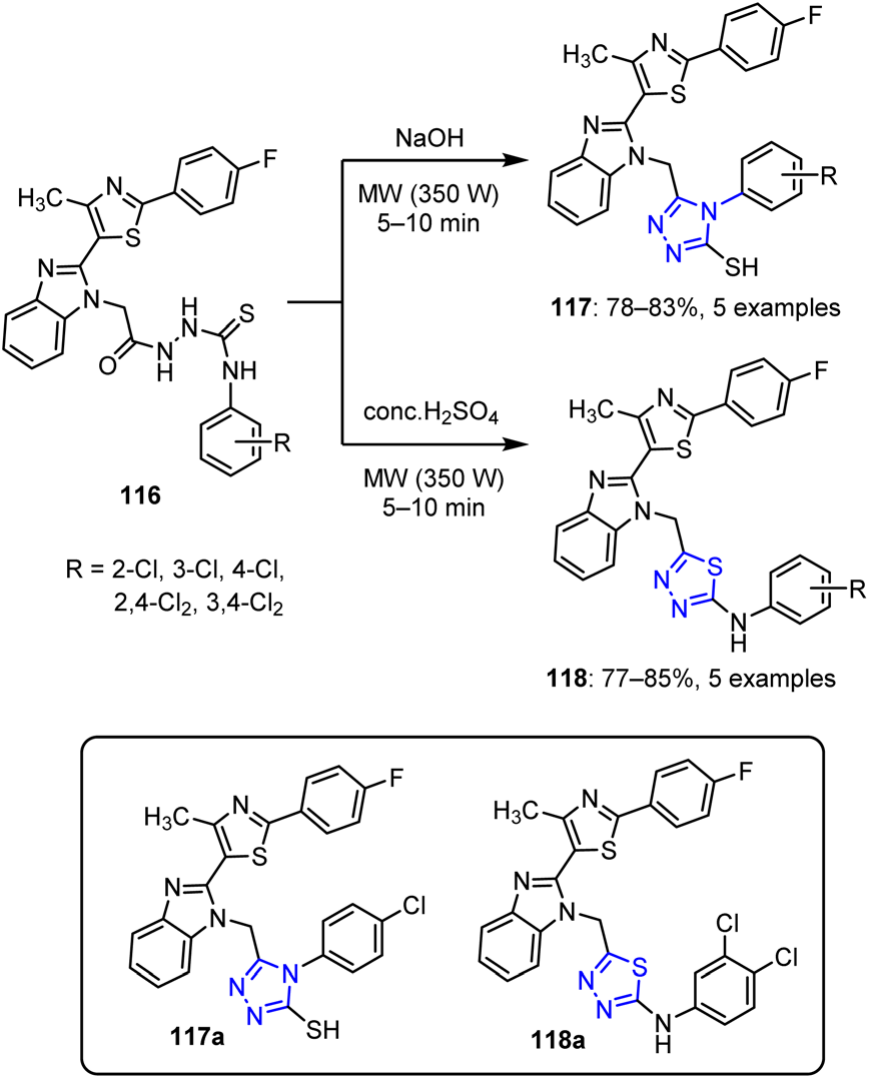
Synthesis of 1,2,4-triazolederivatives 117 and 1,3,4-thiadiazoles 118 from compound 116.

The same authors expanded their research by synthesizing 1,2,4-triazole derivatives 117 and 1,3,4-thiadiazoles 118 from thiosemicarbazide-cored imidazole derivatives 116 (Scheme 29).^94^ When compound 116 was treated with a base under MW irradiation at 350 W for 5–10 min, 1,2,4-triazole derivatives 117 were formed. On the other hand, treating compound 116 with acid under MW conditions yielded 1,3,4-thiadiazoles 118. In the 1,2,4-triazole series, *para*-substituted derivative 117a exhibited a superior activity, while in the 1,3,4-thiadiazole series, both *para* and *meta* substitutions (*e.g.*, compound 118a) led to enhanced activities.

## 4. N-heterocycles with antifungal activities

Antifungal N-heterocycles are nitrogen-containing heterocyclic compounds that inhibit fungal growth by targeting key cellular processes. They act by disrupting cell membranes, inhibiting ergosterol biosynthesis, interfering with DNA/RNA synthesis, or blocking essential fungal enzymes. Their structural diversities and hydrogen bonding abilities enhance their binding to fungal targets, rendering them valuable in the field of antifungal drug development.^95,96^

### 4.1 Microwave-assisted synthesis of anti-fungal compounds

#### 4.1.1 Quinolines

**Scheme 30:**
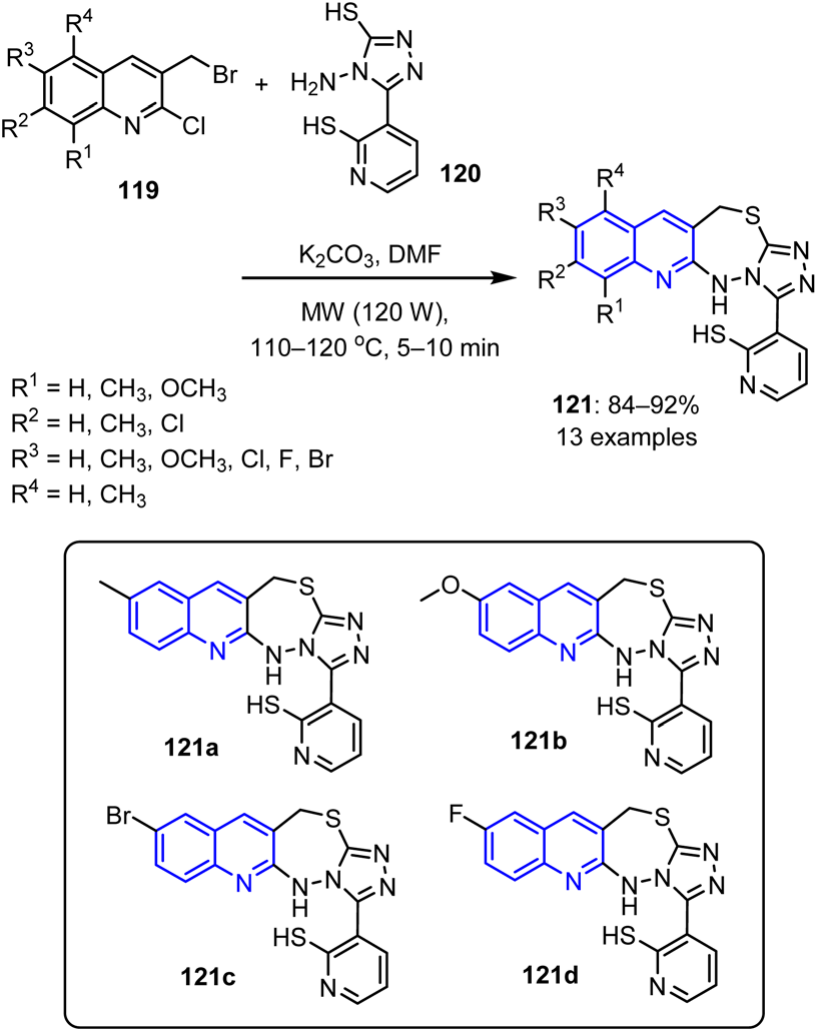
Synthesis of triazolothiadiazepinyl quinolines 121.

Kauser *et al.* reported the reaction of the substituted 4-amino-1,2,4-triazole-3-thiol 120 with 3-(bromomethyl)-2-chloro-quinoline derivatives 119 in DMF.^97^ Subsequent treatment of the reaction mixture with potassium carbonate under MW irradiation at 120 W for 5–10 min led to the formation of triazolothiadiazepinyl quinolines 121 (Scheme 30).

The antiviral properties of the synthesized molecules were subsequently evaluated, revealing that compounds bearing methyl 121a, methoxy 121b, bromo 121c, and fluoro 121d substituents exhibited MIC values. These compounds exhibited significant inhibitory effects against *Aspergillus fumigatus*, indicating their promising antiviral potential.

#### 4.1.2 Triazole

**Scheme 31:**
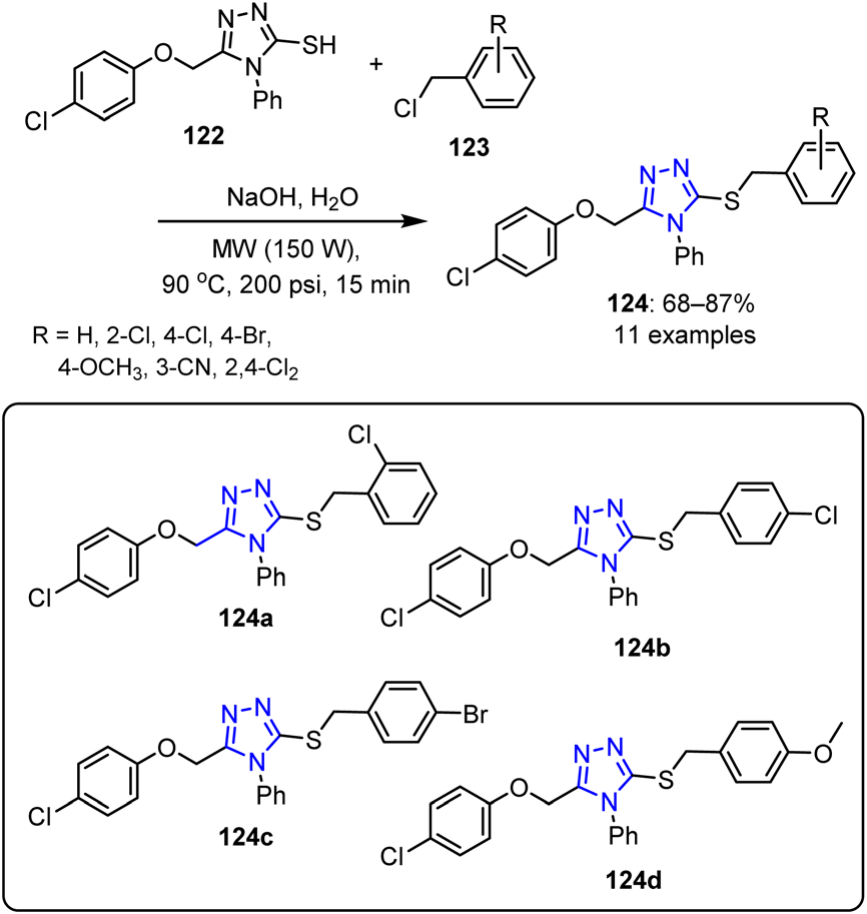
Synthetic procedure for the preparation of novel 1,2,4-triazole-based thioethers 124.

Min *et al.* reported the synthesis of novel 1,2,4-triazole-based thioethers 124.^98^ This approach was based on the reaction of substituted 1,2,4-triazole-3-thiol 122 with substituted benzyl chloride 123 in DMF, employing NaOH as the base. The reaction was conducted under optimized conditions using a CEM Discover Focused Synthesizer, affording the desired thioether derivatives in yields ranging from 68% to 87% (Scheme 31).

Among the synthesized novel 1,2,4-triazole-cored thioether derivatives, the compounds bearing 2-chlorobenzene 124a, 4-chlorobenzene 124b, 4-bromobenzene 124c, and 4-methoxybenzene 124d moieties exhibited strong inhibition against the fungus *Corynespora cassiicola*. Additionally, some of these compounds also demonstrated promising antiviral activities against *Pythium ultimum* Trow.

#### 4.1.3 Pyrrolidine

**Scheme 32:**
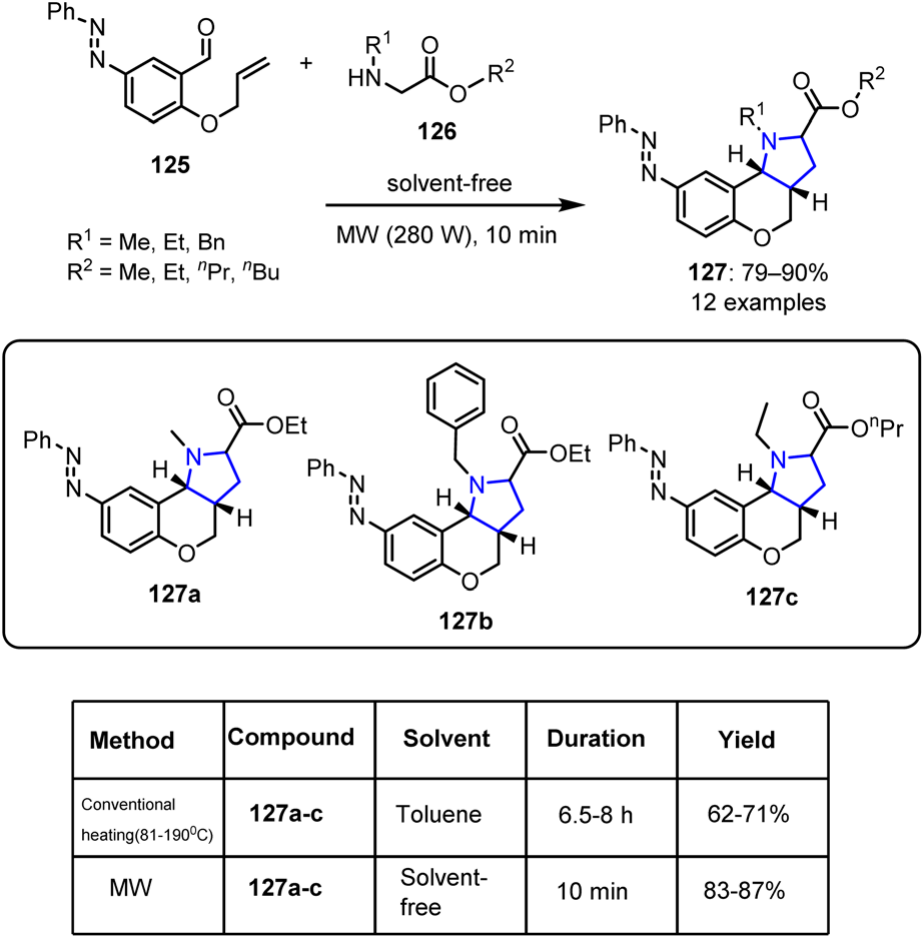
Synthesis of phenyldiazenylchromeno fused pyrrolidines 127.

The intramolecular azomethine ylide cycloaddition of *O*-allyl-5-phenyldiazenylsalicylaldehyde 125 with various esters 126 under MW irradiation produced aryldiazenylchromeno[4,3-*b*]pyrrolidines 127 in yields of 80–90% (Scheme 32).^99^ While some of the synthesized compounds exhibited antibacterial and antitubercular activities, compounds 127a–127c showed exceptional antifungal activities against *Candida albicans*, with MIC values lower than that of the standard drug griseofulvin. Additionally, all synthesized compounds demonstrated moderate-to-strong activities against various bacterial cell lines.

#### 4.1.4 Pyridine

**Scheme 33:**
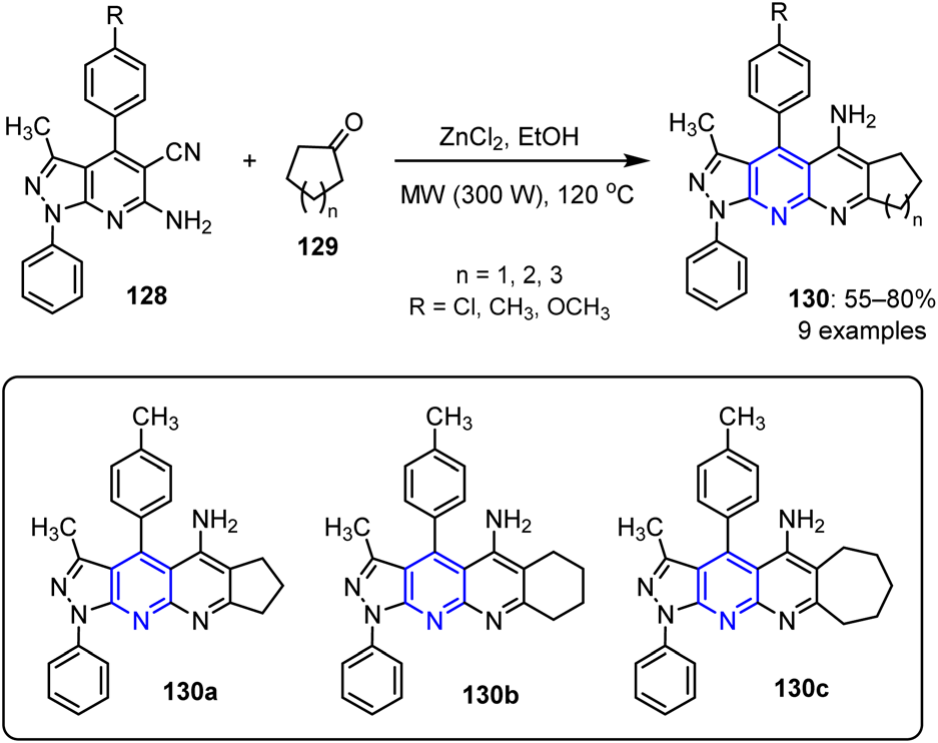
Synthesis of naphthyridine derivatives 130.

Acosta *et al.* reported the synthesis of a novel series of pyrazolo naphthyridin-5-amines 130 using a MW-assisted reaction.^100^ This involved the ZnCl_2_-catalyzed reaction of heterocyclic aminonitriles 128 and cyclic carbonyl compound 129, producing the desired compounds in yields ranging from 55% to 80% (Scheme 33).

**Scheme 34:**
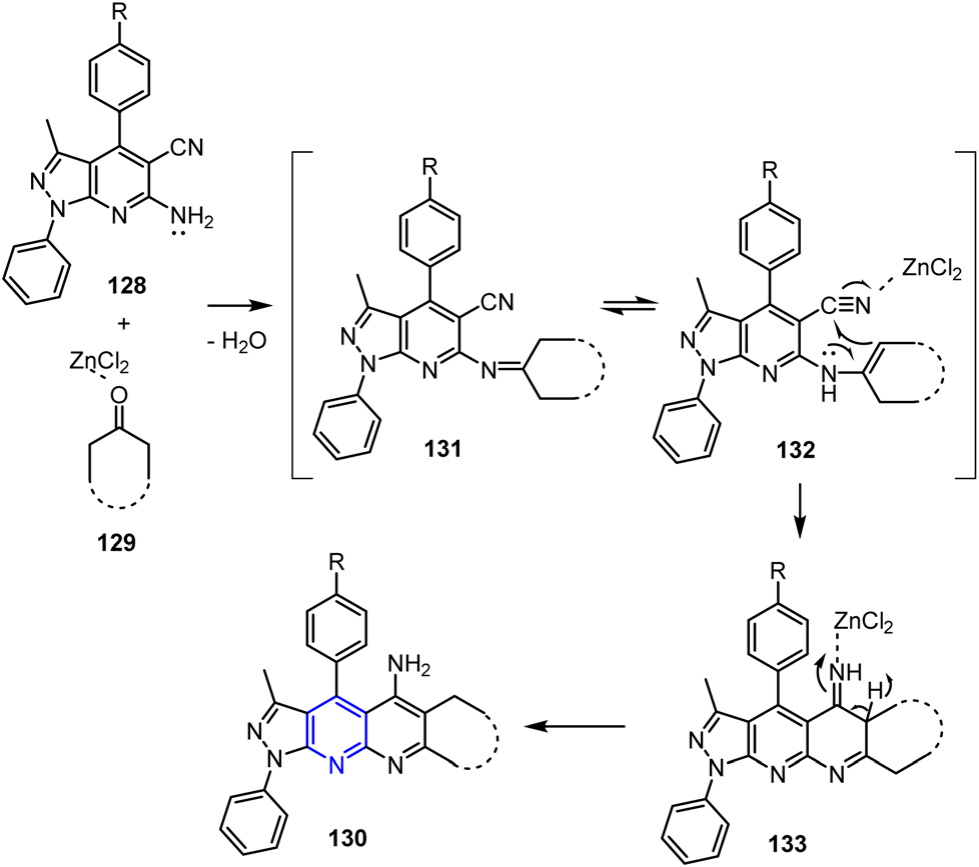
Plausible mechanism for the synthesis of pyrazolo naphthyridin-5-amines 130.

The hypothesized mechanism underlying the cyclization reaction between compounds 128 and 129 involves initial imine formation (intermediate 131) through a typical Friedländer reaction.^101,102^ This intermediate then undergoes an intramolecular nucleophilic attack at the nitrile group, resulting in ring closure and formation of the final product 130. This sequence highlights the efficiency of the Friedländer approach in constructing nitrogen-containing heterocycles (Scheme 34).

**Scheme 35:**
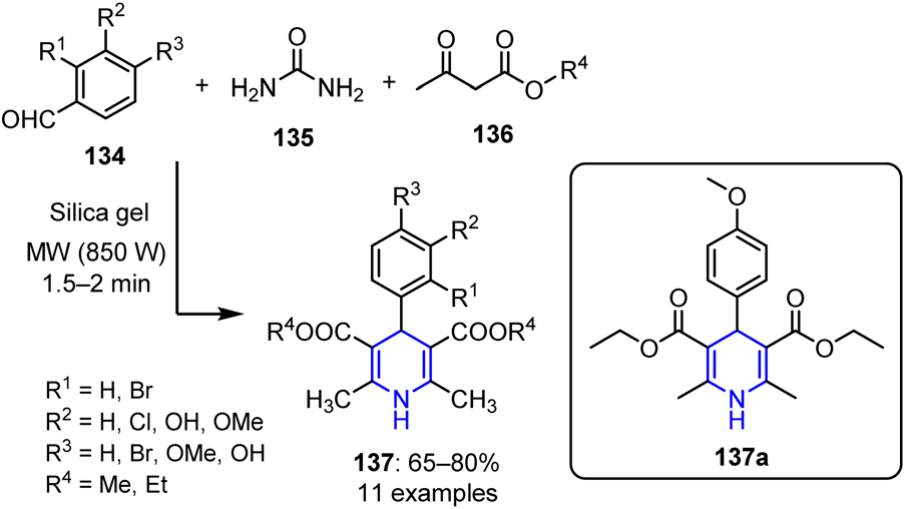
Synthesis of dialkyl-4-aryl-2,6-dimethyl-1,4-dihydropyridin-3,5-dicarboxylates 137.

In 2006, Chhillar *et al.* synthesized novel dialkyl-4-aryl-2,6-dimethyl-1,4-dihydropyridin-3,5-dicarboxylates 137 using the Biginelli cyclocondensation method.^103^ This reaction utilized ethyl/methyl acetoacetate 136, urea 135, and an aromatic aldehyde 134, and was performed under MW irradiation at 850 W for up to 2 min. The desired products were obtained in moderate yields (Scheme 35).

The antifungal activities of all synthesized compounds were subsequently evaluated using the disc diffusion assay, microbroth dilution assay, and percentage spore germination inhibition assay against *A. fumigatus* and *C. albicans*. While all compounds demonstrated notable efficacies, diethyl 4-(4-methoxyphenyl)-2,6-dimethyl-1,4-dihydropyridine-3,5-dicarboxylate 137a exhibited the strongest antifungal activity. Additionally, the colony-forming unit assay confirmed that compound 137a possesses a strong anti-*Candida* action and effectively inhibits the growth of *A. fumigatus*. This suggests that the 4-methoxyphenyl group plays a crucial role in the ability of dihydropyridine to suppress *A. fumigatus* development.

#### 4.1.5 Pyrazole

**Scheme 36:**
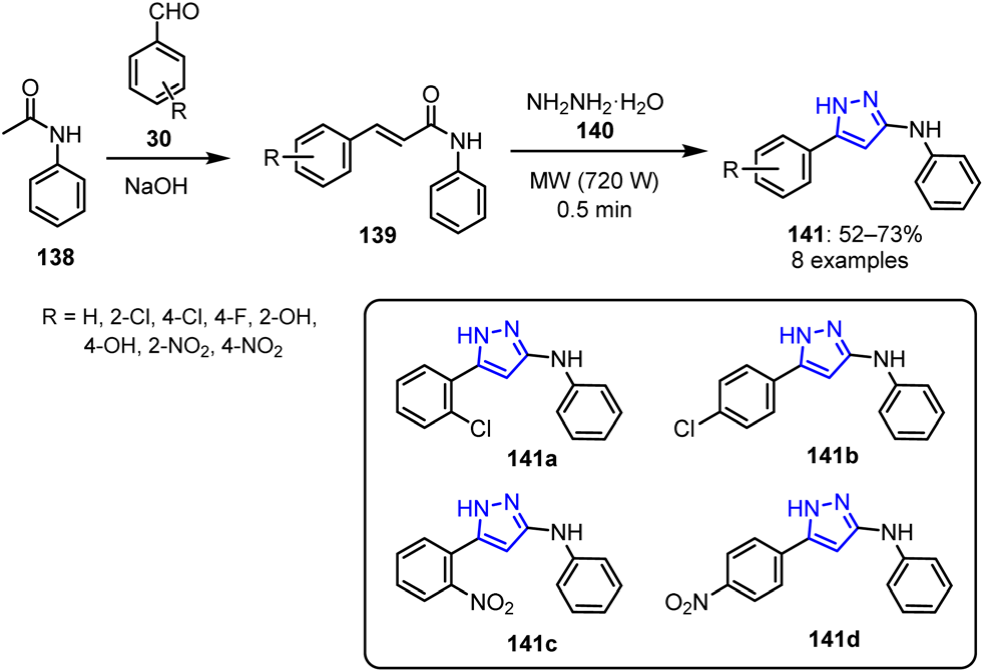
Synthetic route to bioactive pyrazolines 141.

A methanolic solution of benzaldehydes 30 and acetanilide 138 was irradiated at 540 W in the presence of aqueous NaOH to produce various acrylamides 139.^104^ The acrylamides were subsequently treated with hydrazine hydrate 140 in the presence of glacial acetic acid under MW irradiation at 720 W, resulting in the formation of the corresponding pyrazolines 141 with good yields ranging from 52% to 73% (Scheme 36).

The antifungal activities of all synthesized pyrazolines were evaluated using the poisoned food technique, with Bavistin as the standard drug. The compounds bearing chloro substituents at the *ortho*141a and *para*141b positions exhibited ED_50_ values of 930 and 760 μg mL^−1^ against *D. maydis*, respectively. These compounds showed inhibition comparable to Bavistin at 1000 μg mL^−1^ against *R. solani*. Compounds with nitro groups 141c and 141d also demonstrated similar inhibition activities at all concentrations.

## 5. N-heterocycles with antimicrobial activities

N-heterocyclic compounds exhibit remarkable antimicrobial properties by targeting key pathways that are essential for microbial survival and growth. Their effectiveness against a broad spectrum of microbial pathogens is attributed to their ability to disrupt essential biological processes, including cell wall synthesis, protein formation, and DNA replication.^105–107^

### 5.1 Microwave-assisted synthesis of antimicrobial agents

#### 5.1.1 Pyrrole

**Scheme 37:**
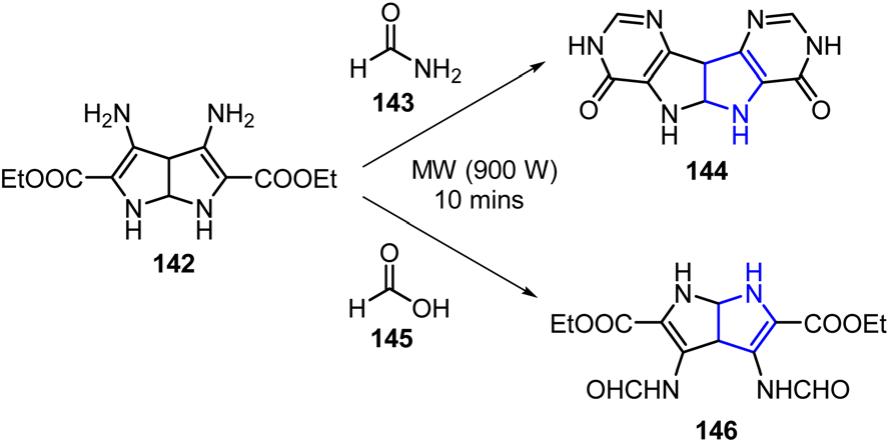
MW-assisted synthesis of novel pyrrolo[2,3-*b*]pyrrole derivatives 144 and 146.

Moumen *et al.* developed a MW-assisted synthesis of novel pyrrolo[2,3-*b*]pyrrole derivatives 144 and 146 using formamide 143 and formic acid 145 as substituents, affording the desired compounds in appreciable yields (Scheme 37).^108^ The synthesized compounds were subsequently evaluated for their biological activities. Several derivatives exhibited moderate-to-good antimicrobial activities. For example, compound 144 showed a moderate efficacy against *Pseudomonas aeruginosa* (MIC: 50 μg mL^−1^), although it was less potent than ciprofloxacin (MIC: 25 μg mL^−1^). Additionally, compound 146 exhibited a significant activity against *S. aureus*, showing effectiveness comparable to that of ciprofloxacin, though it was approximately half as potent as ampicillin. Meanwhile, compound 144 demonstrated an antifungal activity against *C. albicans* that reached approximately 25% that exhibited by clotrimazole, while also demonstrating the highest antioxidant capacity with a 59% radical scavenging activity. Additionally, the majority of the synthesized derivatives displayed encouraging anticancer activities across the three evaluated cell lines.

## 6. N-heterocycles with anti-inflammatory activities

N-heterocyclic compounds are key scaffolds in anti-inflammatory drug development since their nitrogen atoms promote interactions with biological targets, such as COX-2 and the cytokine receptors. Additionally, their structural versatility allows for strong binding, good bioavailabilities, and superior metabolic stabilities, often resulting in improved efficacies and reduced toxicities compared with those of non-heterocyclic compounds.^109,110^

### 6.1 Microwave-assisted synthesis of anti-inflammatory agents

#### 6.1.1 Imidazole

**Scheme 38:**
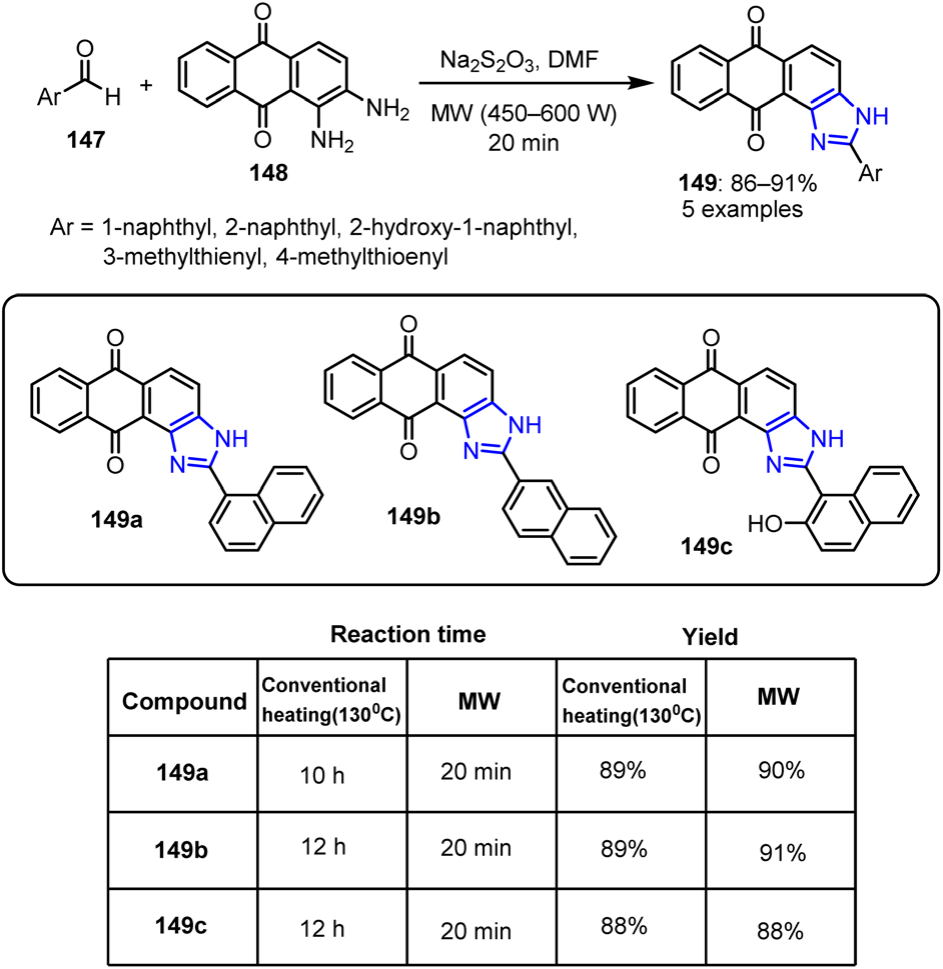
Synthesis of 3-aryl-anthra[1,2-*d*] imidazole-6,11-diones 149.

Sondhi *et al.* reported a series of imidazole derivatives with promising potential for exhibiting enhanced anti-inflammatory and anticancer activities. The cyclization of aromatic aldehydes 147 with 1,2-diaminoanthraquinone 148 was carried out under MW irradiation at 450 W for 5 min.^111^ Using sodium metabisulfite and DMF as catalysts, this reaction yielded the corresponding 3-aryl-anthra[1,2-*d*]imidazole-6,11-diones 149 (Scheme 38).

All synthesized compounds were evaluated for their anti-inflammatory and anticancer activities using the carrageenan-induced paw edema model. Ibuprofen, which was used as the reference drug, displayed the highest anti-inflammatory effect at approximately 39% inhibition with an oral dose of 50 mg kg^−1^. Among the tested compounds, the compound bearing a 2-naphthalenyl substituent (149b) exhibited an anti-inflammatory activity that was nearly equivalent to that of ibuprofen. In contrast, 1-naphthalenyl-substituted derivatives 149a and 149c exhibited enhanced anticancer activities.

**Scheme 39:**
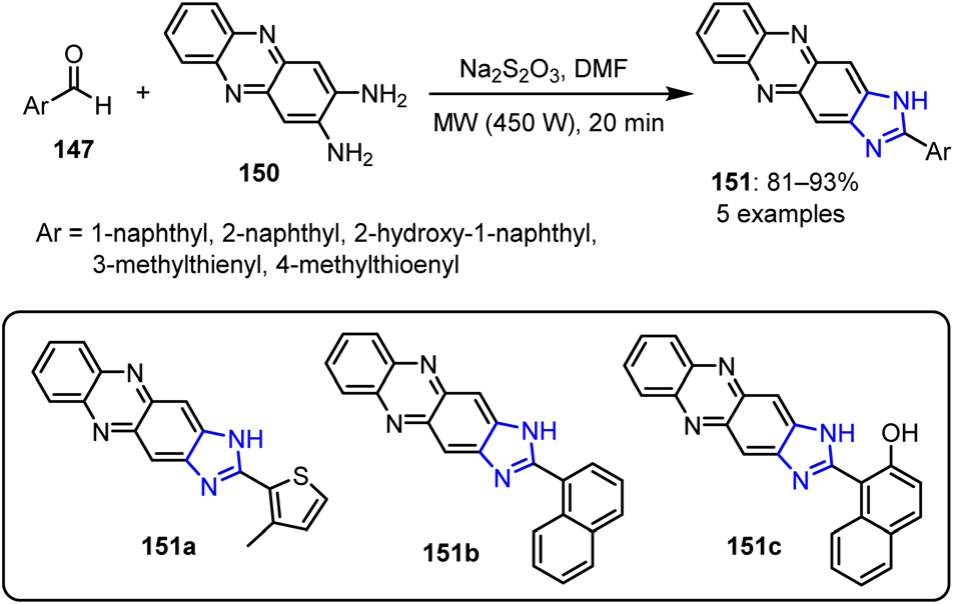
Synthesis of 1-aryl-imidazo[4,5-*b*] phenazine 151.

Another series of imidazole derivatives 151 was synthesized under the same conditions, using phenazine-2,3-diamine 150 instead of 1,2-diaminoanthraquinone 148 as the starting material (Scheme 39). Similar to the previous series, naphthalenyl-substituted derivatives 151b and 151c demonstrated anticancer activities, while compound 151a exhibited a 38% anti-inflammatory activity when administered orally at a dose of 50 mg kg^−1^.

**Scheme 40:**
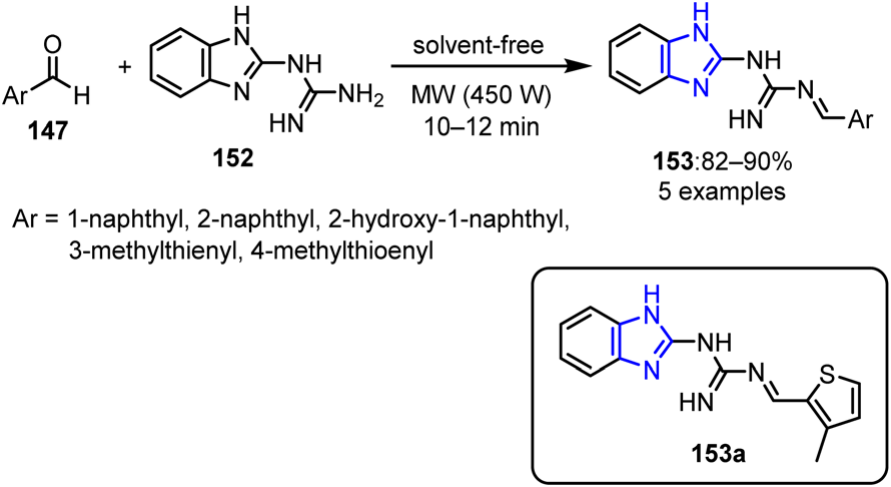
Synthesis of 1-(1*H*-benzo[*d*]imidazol-2-yl)-3-((aryl) methylene)guanidines 153.

The authors further extended their research by synthesizing imidazole-cored guanidine derivatives 153.^111^ This synthetic approach employed 2-guanidinobenzimidazole 152 and aromatic aldehyde 147 as starting reagents. The reactions were conducted without the use of solvent, utilizing MW irradiation at 450 W for 10–20 min to yield the target products with yields up to 90% (Scheme 40). The imidazole-based guanidine structure bearing a thiophene derivative 153a exhibited promising anti-inflammatory properties comparable to those of ibuprofen. However, no other compounds in this series showed anticancer activity.

### 6.2 Sonochemical synthesis of anti-inflammatory agents

#### 6.2.1 Indole

**Scheme 41:**
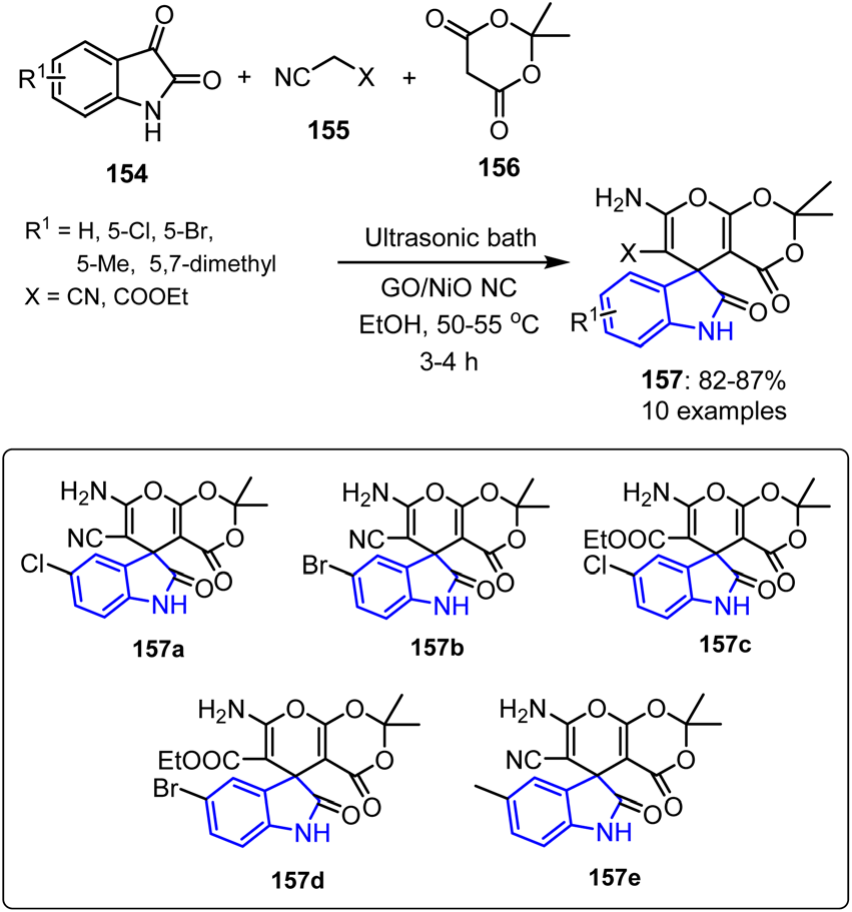
Synthesis of spiro[indoline-3,5′-pyrano[2,3-*d*]-[1′3′]dioxine] derivatives 157.

Khushboo *et al.* established an efficient synthetic approach for the preparation of spiroindole derivatives 157, achieving yields between 84% and 87% (Scheme 41). This method employed a catalytic quantity of a graphene oxide-supported nickel oxide nanocomposite (GO/NiO NC) as a highly active heterogeneous catalyst under ultrasonic irradiation. Remarkably, the GO/NiO NC catalyst exhibited a good recyclability, retaining its catalytic efficiency over three successive cycles with only a minor reduction in activity.^112^

The synthesized spiroindole derivatives, particularly compounds 157a–157e, demonstrated significant anti-inflammatory activities by effectively preventing protein denaturation at all tested concentrations, indicating their potential for therapeutic use. Specifically, compound 157b exhibited the strongest anti-inflammatory effect, with inhibition rates ranging from 43.53% to 55.75% at concentrations of 20–100 mg L^−1^. This compound also displayed the lowest IC_50_ value (55.85 mg L^−1^), rendering it the most potent compound in the series.

**Scheme 42:**
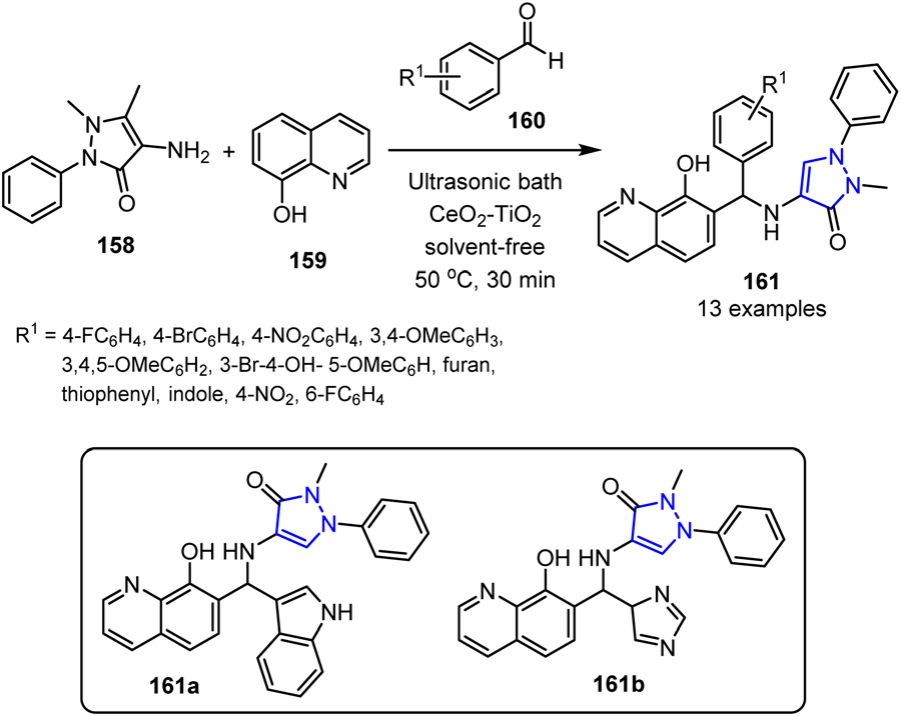
CeO_2_–TiO_2_-catalyzed synthesis of antipyrine-linked quinolines 161.

In another study, Bhaskar *et al.* developed an efficient, eco-friendly, and straightforward method for synthesizing bioactive antipyrine-linked quinoline analogues 161 using a recyclable CeO_2_–TiO_2_ nanocatalyst and ultrasonic irradiation.^113^ This green approach leverages the synergistic effects of the nanocatalyst and ultrasound-assisted multicomponent reactions, enabling rapid hybrid construction with high yields under solvent-free conditions, thereby saving time, energy, and costs. The antibacterial and antioxidant activities of the synthesized compounds were subsequently assessed, with 161a and 161b demonstrating notable potencies in both cases (Scheme 42). Compared to conventional catalysts, the biogenically synthesized CeO_2_–TiO_2_ nanocatalyst offers a superior performance, delivering higher yields in shorter reaction times, with an excellent reusability and minimal leaching. These findings underscore the potential of antipyrine hybrids for use as valuable frameworks in the development of novel pharmaceutical agents.

#### 6.2.2 Quinoline

**Scheme 43:**
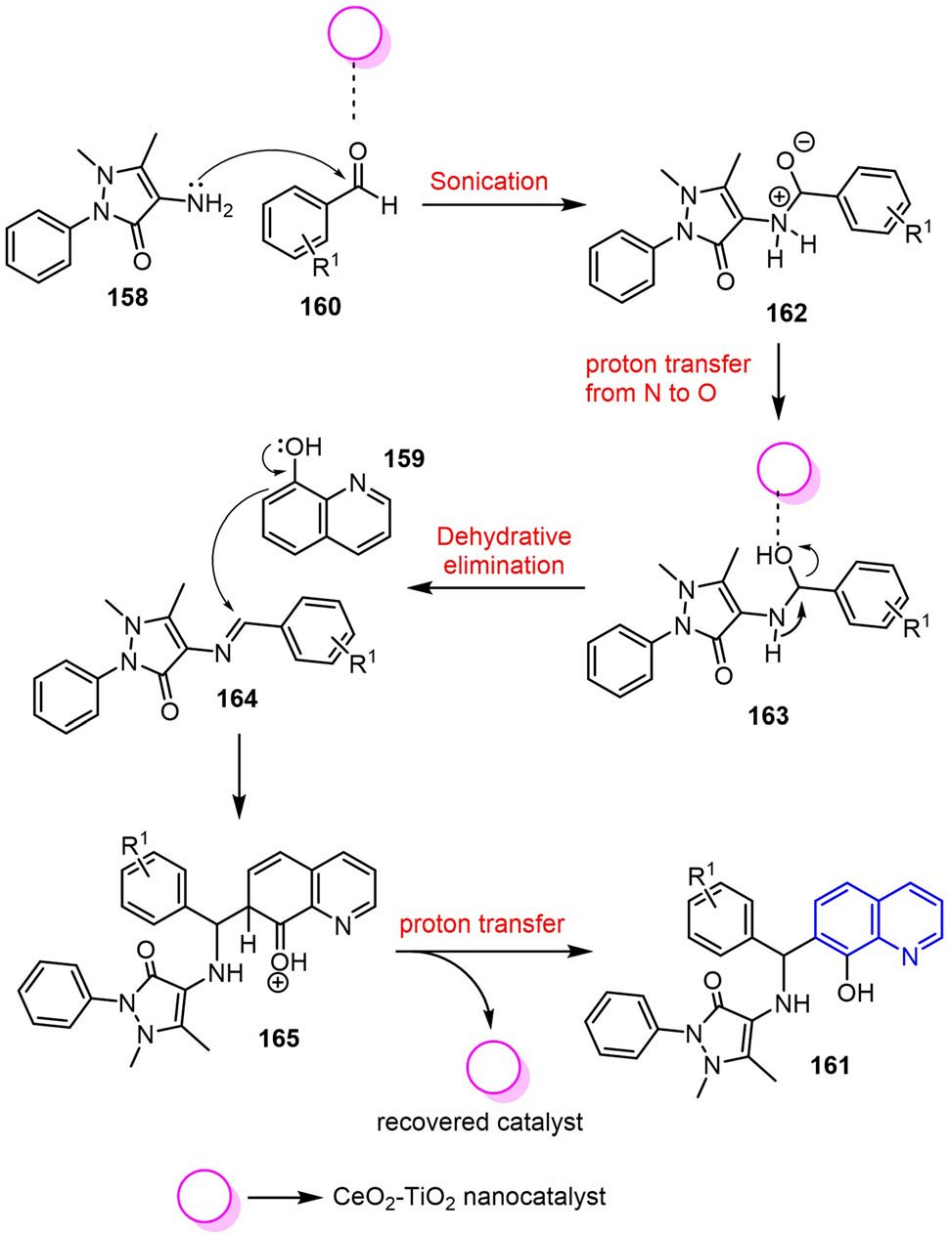
Plausible reaction mechanism for the CeO_2_–TiO_2_ nanocatalyzed synthesis of quinoline analogues 161.

The synthesis of quinoline derivatives 161 involved a CeO_2_–TiO_2_ nanocatalyst-mediated activation of aromatic aldehydes 160, which increased the electrophilicity of the carbonyl carbon. This facilitated condensation with 4-aminoantipyrine 158, followed by proton transfer and dehydration to form a Schiff base intermediate 163.^114,115^ Subsequently, this intermediate reacted with 8-hydroxyquinoline 159, undergoing cyclization to yield the target compounds 161 (Scheme 43).

In this reaction, the nanocatalyst plays a dual role, initially activating the aldehyde, and later deprotonating the quinoline hydroxyl group, ultimately enhancing the nucleophilicity and accelerating Schiff base formation. This promotes a faster, solvent-free reaction with a high efficiency, and the nanocatalyst can be easily recovered and reused, thereby supporting the principles of green synthesis.

## 7. N-heterocycles with antitubercular activities

Nitrogen-containing ring-structured N-heterocyclic compounds have been demonstrated to exhibit potent anti-mycobacterial action. Specifically, scaffolds such as triazoles, pyridines, and quinolines interfere with various bacterial processes, including DNA replication and cell wall construction. Two important examples of such compounds are bedaquiline and isoniazid. The potencies and structural adaptabilities of these substances render them essential for the development of TB drugs.^116,117^

### 7.1 Sonochemical synthesis of antitubercular agents

#### 7.1.1 Pyrazole

**Scheme 44:**
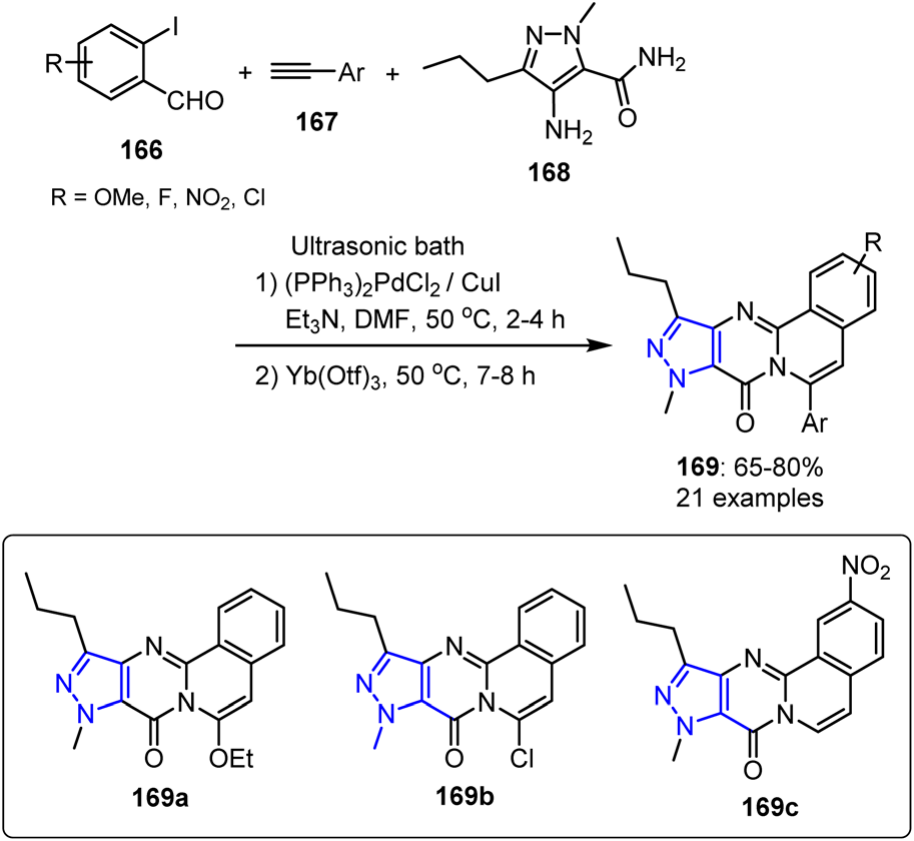
Ultrasound irradiation assisted one-pot synthesis of 6-arylpyrazolo[4,3:4,5]pyrimido[2,1-*a*]isoquinolin-8(9*H*)-ones 169.

Venkateswara *et al.* developed a synthetic protocol for a series of molecules containing the 4-amino-1-methyl-3-propyl-1*H*-pyrazole-5-carboxamide fragment 168 as potential chorismate mutase (CM) inhibitors.^118^ This approach employed a Sonogashira coupling reaction between 2-iodobenzaldehydes 166 and a terminal alkyne 167, generating 2-alkynylbenzaldehyde intermediates *in situ*. These intermediates subsequently underwent Yb(iii)-catalyzed sequential triazole reactions with 4-amino-1-methyl-3-propyl-1*H*-pyrazole-5-carboxamide 168, affording the target compounds in moderate-to-good yields. This strategy demonstrated a broad tolerance for different substituents on both the terminal alkynes (OMe, OEt, Me, Et, ^*n*^Pr, ^*n*^Bu, and Cl) and the aromatic aldehydes (OMe, F, NO_2_, Me, and Cl). The approach was further applied to synthesize 6-aryl-8*H*-isoquinolino[1,2-*b*]quinazolin-8-one derivatives. Subsequently, the prepared compounds were evaluated through molecular docking at the MtbCM binding site (PDB 2FP2). Based on the favorable docking outcomes, the researchers performed an ultrasound-assisted one-pot synthesis to produce 6-arylpyrazolo[4,3:4,5]pyrimido[2,1-*a*]isoquinolin-8(9*H*)-one derivatives 169 (Scheme 44).

Three derivatives, namely 169a–169c, which exhibited strong *in silico* interactions with CM, demonstrated significant *in vitro* inhibition efficiencies (60–67%) at 10 μM. SAR analysis showed that attaching an NO_2_ group to the C-2 position of the core structure significantly boosted the potency of the active species, while the introduction of OMe and F groups at the same position resulted in moderate and low activities, respectively. Aryl substituents, such as *p*-methoxyphenyl and *p*-chlorophenyl groups at the C-6 position, were beneficial for promoting the antitubercular activity, in contrast to *p*-alkoxy and *p*-alkyl phenyl groups, which were less effective. Compound 169c demonstrated a promising antitubercular potential in preliminary evaluations, supported by its MtbCM inhibitory activity, favorable docking results, and predictive absorption, distribution, metabolism, and excretion (ADME) properties.

## 8. N-heterocycles with antiviral activities

N-heterocycles are a cornerstone in antiviral research due to their structural variety and significant biological activities. These nitrogen-containing rings interfere with viral propagation, enzyme activities, and entrance into host cells. At present, they continue to inspire innovative treatments against prevalent and emerging viruses, and are present in many licensed medications.^119,120^

### 8.1 Sonochemical synthesis of antiviral agents

#### 8.1.1 Quinazoline

**Scheme 45:**
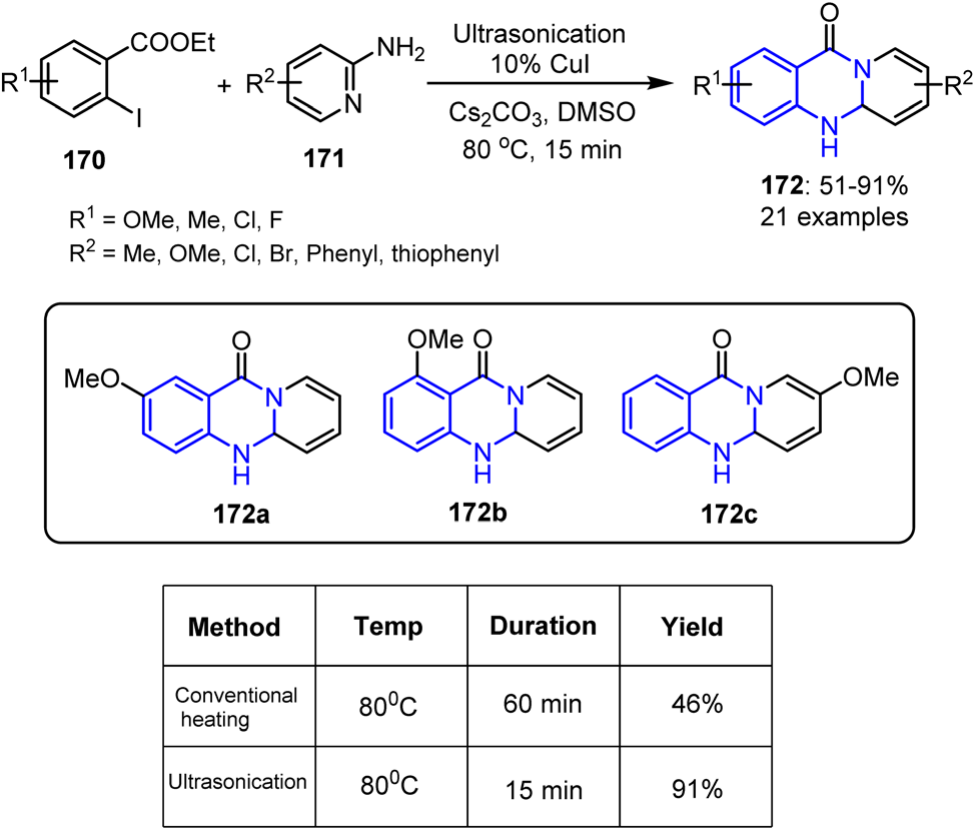
Copper-catalyzed one-pot synthesis of 11*H*-pyrido[2,1-*b*]quinazolin-11-ones 172 facilitated by ultrasonic irradiation.

Matta *et al.* reported a novel synthetic strategy for the preparation of 11*H*-pyrido[2,1-*b*]quinazolin-11-one derivatives 172 (Scheme 45). Their route involved a CuI-catalyzed Ullmann–Goldberg coupling followed by a one-pot cyclization reaction under ultrasonic irradiation, affording the target products in moderate-to-good yields. Utilizing 2-iodobenzoate ester 170 in combination with 2-aminopyridine 171, quinolin-2-amine, or thiazol-2-amine also led to successful formation of the desired compounds. Additionally, an *in silico* study was conducted to evaluate the interaction of these derivatives with the SARS-CoV-2 RNA-dependent RNA polymerase (RdRp).^121^

**Scheme 46:**
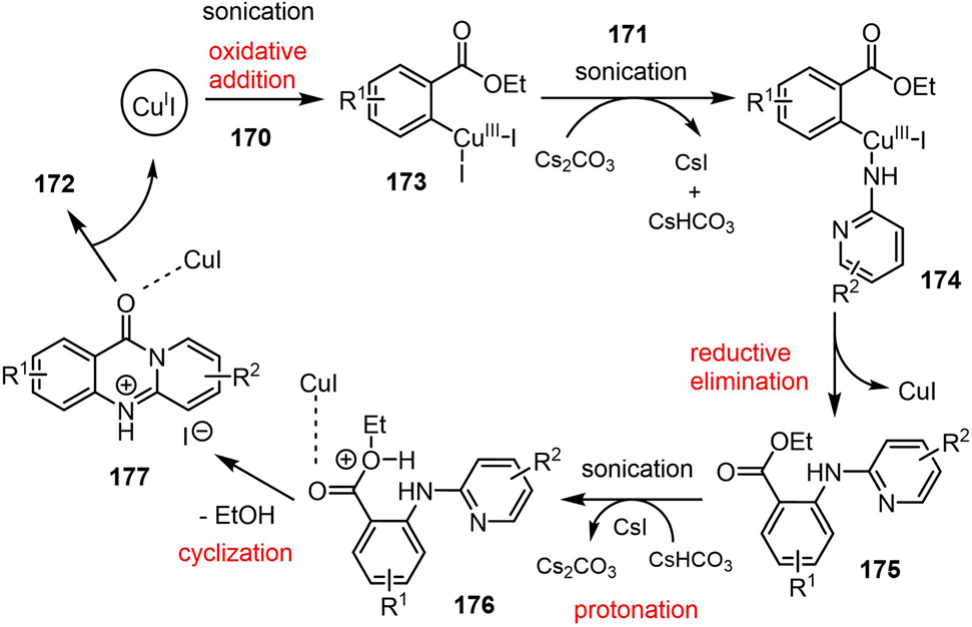
Plausible mechanism for the sonochemical synthesis of 11*H*-pyrido[2,1-*b*]quinazolin-11-one derivatives 172.

This copper-catalyzed, ultrasound-assisted reaction was initiated by the oxidative addition of CuI to the 2-iodobenzoate ester 170, forming an intermediate 173 that reacts with 2-aminopyridine 171 to give a Cu-amidate complex 174. Reductive elimination subsequently yields a key intermediate 175, which undergoes intramolecular cyclization *via* ester activation, forming 11*H*-pyrido[2,1-*b*]quinazolin-11-one 172. Alternatively, CuI may initially bind to 2-aminopyridine before coupling, thereby generating the same product (Scheme 46).

Molecular docking studies targeting SARS-CoV-2 RdRp (PDB ID 7AAP) revealed that compounds 172a and 172c exhibited similar interaction profiles, while 172b showed a binding efficiency that was nearly equivalent to those of favipiravir and remdesivir. Specifically, 172a formed two hydrogen bonds, namely between its methoxy (OMe) group and THR556, and between its N-5 atom and SER682. Additionally, compound 172b established hydrogen bonds between its OMe group and ARG624, and between its carbonyl (C=O) group and THR556. Compound 172c, which demonstrated the most favorable binding energy, formed hydrogen bonds between its OMe group and THR556 and ARG624, and also between its N-5 atom and SER682. Pharmacokinetic evaluations indicated that all three compounds possessed favorable ADME properties, with the exception of their blood–brain barrier permeabilities. The work of Matta *et al.* therefore not only provides a CuI-catalyzed, ultrasound-assisted route to 11*H*-pyrido[2,1-*b*]quinazolin-11-one derivatives, but it also underscores the potential of these compounds for use as promising SARS-CoV-2 RdRp inhibitors.

#### 8.1.2 Isoquinoline

**Scheme 47:**
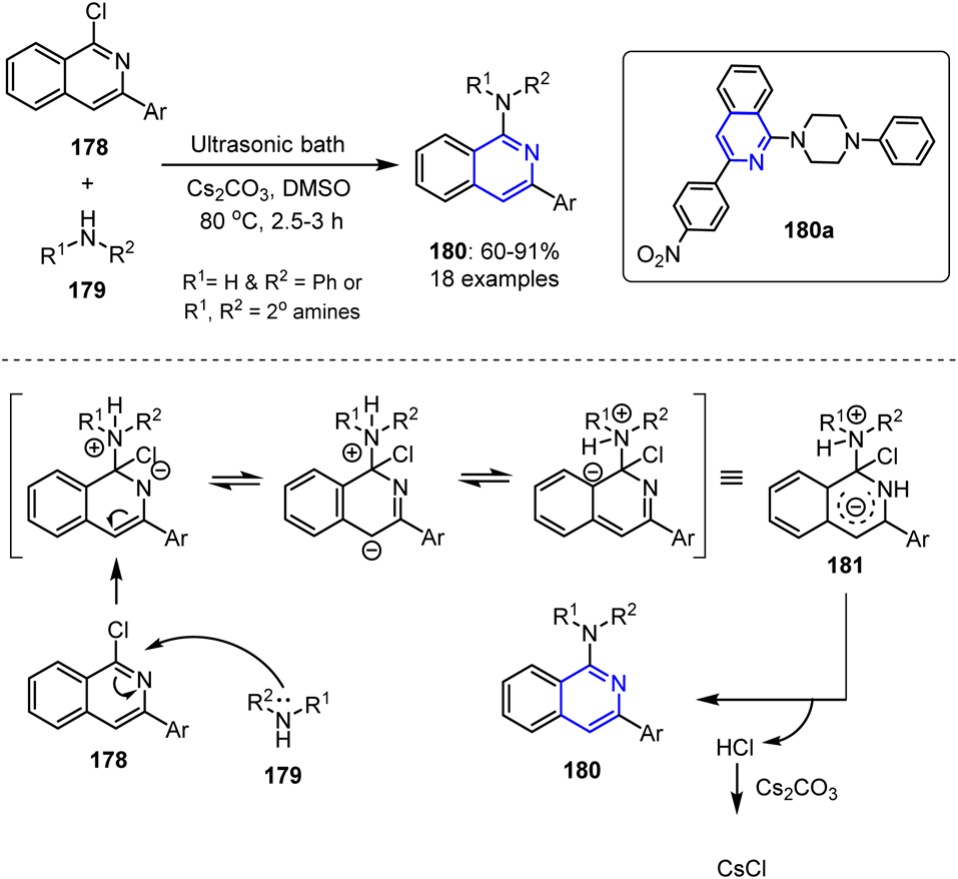
Sonochemical synthesis of 1-aminoisoquinoline derivatives 180 through the reaction of 1-chloroisoquinoline 178 with appropriate amines 179.

Daliparthi *et al.* reported the synthesis of 1-aminoisoquinolines 180 under sonochemical conditions by treating 1-chloroisoquinoline 178 with different amines 179 in the presence of Cs_2_CO_3_ and DMSO (Scheme 47).^122^ This reaction began with an ultrasound-assisted nucleophilic attack by amine 179 on the chlorine-bearing C-1 atom of aromatic reactant 178, forming a resonance-stabilized intermediate 181. Ultrasonic irradiation facilitated both the nucleophilic substitution and the subsequent elimination of HCl from 181, which restored aromaticity and efficiently yielded the final product 180.

The same group further explored the antiviral potential of 1-aminoisoquinoline derivatives against the NS5 protein of the dengue virus (DENV) through *in silico* docking studies. Since the MTase domain of the NS5 protein is involved in RNA capping and the RdRp domain plays a key role in viral replication, both were targeted during their work. Docking studies with the RdRp domain (PDB 5I3Q) revealed that compound 180a forms strong hydrogen bonds with the GLN802, LEU511, and HIS512 residues through its NO_2_ group. Similarly, docking into the DENV3 MTase domain (PDB 5EHG) showed that the same compound formed three hydrogen bond interactions with the GLY86, TRP87, and SER56 residues, highlighting its promising antiviral potential.

## 9. Conclusions

In conclusion, although non-conventional synthetic techniques, such microwave- and ultrasound-assisted procedures have numerous advantages, they also exhibit various limitations. Specifically, the requirement for specialized equipment, which may raise initial costs and necessitate technical expertise, is one of the main disadvantages. These techniques may also be less scalable, thereby rendering them less appropriate for use in large-scale industrial manufacturing. An inconsistent product quality may also result from the possibility of hot patches or uneven heating during microwave synthesis. Furthermore, ultrasonic bath reactions frequently suffer from poor reproducibilities due to differences in the bath layouts and a lack of standardized control over the ultrasonic power. Despite these challenges, there are numerous benefits to non-conventional approaches. For example, these methods greatly shorten the reaction times, increase yields, and lead to improved selectivities compared with those achieved using conventional synthetic methods. Moreover, through the use of reduced solvent volumes and lower extents of energy consumption, they also provide more environmentally friendly and energy-efficient alternatives. Consequently, non-conventional routes tend to be more efficient, sustainable, and reproducible than traditional approaches, which is particularly significant for the rapid and effective synthesis of bioactive molecules.

In this review, the preparation of a range of N-heterocyclic compounds using non-conventional synthetic techniques was examined. The findings of this review suggest that such approaches offer numerous advantages, including higher yields, a reduced environmental impact, and enhanced selectivities. The biological activities of the synthesized N-heterocyclic compounds were subsequently evaluated, revealing noteworthy potential in various applications due to their antimicrobial, anticancer, anti-inflammatory, and antiviral activities. Microwave-assisted routes yielded compounds with enhanced potencies and selectivities, highlighting their potential for use as drug candidates in diverse therapeutic applications.

Overall, this review highlights the significance of non-traditional synthetic approaches as effective and sustainable strategies for the production of biologically active N-heterocyclic compounds. The presented results pave the way for new opportunities in drug discovery and in the development of novel pharmaceuticals. Nonetheless, further work in this field should concentrate on enhancing the reproducibility, including through the use of continuous-flow and extrusion technologies, along with the integration of artificial intelligence and *in situ* monitoring. These techniques will be expected to provide greener, faster, and more efficient alternatives to traditional processes, thereby playing an important role in sustainable chemical production.

## 10. Future development on non-conventional synthesis techniques

### 10.1 Microwave assisted synthesis (MAOS)

Anticipated advancements in microwave-assisted synthesis are likely to transition from fixed-frequency setups to variable-frequency and tailored field irradiation, facilitating more precise control over energy distribution. The design of reactors will emphasize the development of rational catalyst supports, microwave-transparent flow cells, and hybrid technologies that integrate microwave energy with induction heating, ultrasound, or photo redox activation. Furthermore, the incorporation of digital twin models is expected to enable predictive design and reliable scale-up, thereby mitigating the current challenges of non-uniform heating and poor reproducibility.

### 10.2 Sonochemical synthesis

The future of sonochemistry will predominantly focus on the utilization of multi-frequency, pulsed, and chirped ultrasonic waves to achieve programmable cavitation. This approach will facilitate precise control over bubble collapse and radical synthesis. Additionally, it is anticipated that hybrid systems, such as sonophotocatalysis and sonoelectrochemistry, will become prevalent for complex oxidation processes and selective reactions. These systems will be enhanced by specially engineered reactor surfaces that concentrate cavitation effects at catalyst interfaces.

### 10.3 Mechanochemical synthesis

Mechanochemistry is expected to progress beyond traditional ball milling techniques, advancing towards resonant acoustic mixing and continuous, recipe-controlled extrusion. These developments will offer scalable methods for the preparation of complex materials and active pharmaceutical ingredients (APIs). The integration of *operando* multi-modal monitoring technologies, such as torque measurement, acoustic sensors, and Raman spectroscopy, will facilitate real-time management and control over polymorph selectivity. Furthermore, mechano-biocatalysis and biomass upgrading present promising opportunities for sustainable processes within green chemistry. Specifically designed tribo- and piezo catalysts are anticipated to enhance the utilization of mechanical energy.

The progression of these non-conventional methodologies will depend on the establishment of standardized process analytical technologies (PAT), the integration of real-time sustainability metrics and life-cycle assessments, and the implementation of unified reporting protocols to ensure reproducibility. Artificial intelligence and machine learning are anticipated to play a pivotal role in optimizing reaction conditions by correlating waveform, field, or mechanical inputs with desired outcomes. Additionally, the development of modular hybrid systems that integrate mechanical, acoustic, microwave, photochemical, and electrochemical methodologies will enhance the synthetic repertoire. Collectively, these innovations will facilitate reproducible, scalable, and environmentally sustainable chemical transformations that exceed the limitations of traditional synthetic strategies.

## Author contributions

N. S.: writing—original draft; K. S.: writing—original draft; K. K.: resources, writing—original draft; M. D. K.: writing—original draft; T. D.: conceptualization, funding acquisition, resources, supervision, writing—review and editing; F. V. S.: conceptualization, project administration, supervision, writing—review and editing.

## Conflicts of interest

We declare we have no competing interests.

## Data Availability

No new data was created or analyzed during this study. Therefore, data sharing is not applicable.
